# Structural and Functional Characterization of a *Caenorhabditis elegans* Genetic Interaction Network within Pathways

**DOI:** 10.1371/journal.pcbi.1004738

**Published:** 2016-02-12

**Authors:** Benjamin Boucher, Anna Y. Lee, Michael Hallett, Sarah Jenna

**Affiliations:** 1 Department of Chemistry, Université du Québec à Montréal, Montréal, Québec, Canada; 2 Pharmaqam, Université du Québec à Montréal, Montréal, Québec, Canada; 3 Biomed, Université du Québec à Montréal, Montréal, Québec, Canada; 4 McGill Centre for Bioinformatics, McGill University, Montréal, Québec, Canada; 5 School of Computer Science, McGill University, Montréal, Québec, Canada; 6 Rosalind and Morris Goodman Cancer Centre, McGill University, Montréal, Québec, Canada; University of Minnesota, UNITED STATES

## Abstract

A genetic interaction (GI) is defined when the mutation of one gene modifies the phenotypic expression associated with the mutation of a second gene. Genome-wide efforts to map GIs in yeast revealed structural and functional properties of a GI network. This provided insights into the mechanisms underlying the robustness of yeast to genetic and environmental insults, and also into the link existing between genotype and phenotype. While a significant conservation of GIs and GI network structure has been reported between distant yeast species, such a conservation is not clear between unicellular and multicellular organisms. Structural and functional characterization of a GI network in these latter organisms is consequently of high interest. In this study, we present an in-depth characterization of ~1.5K GIs in the nematode *Caenorhabditis elegans*. We identify and characterize six distinct classes of GIs by examining a wide-range of structural and functional properties of genes and network, including co-expression, phenotypical manifestations, relationship with protein-protein interaction dense subnetworks (PDS) and pathways, molecular and biological functions, gene essentiality and pleiotropy. Our study shows that GI classes link genes within pathways and display distinctive properties, specifically towards PDS. It suggests a model in which pathways are composed of PDS-centric and PDS-independent GIs coordinating molecular machines through two specific classes of GIs involving pleiotropic and non-pleiotropic connectors. Our study provides the first in-depth characterization of a GI network within pathways of a multicellular organism. It also suggests a model to understand better how GIs control system robustness and evolution.

## Author Summary

Network biology has focused for years on protein-protein interaction (PPI) networks, identifying nodes with central structural functions and modules associated to bioprocesses, phenotypes and diseases. Network biology field moved to a higher level of abstraction, and started characterizing a less intuitive kind of interactions, called genetic interactions (GIs) or epistasis. Mostly due to technical challenges associated to the genome-wide mapping of GIs, these studies primarily focused on unicellular organisms. They uncovered modules embedded within the structure of these networks and started characterizing their relationship with PPI-network and biological functions. We provide here the first in-depth characterization of a network composed of ~600 GIs within signaling and metabolic pathways of a multicellular organism, the nematode *Caenorhabditis elegans*. We characterize the structure of this network, and the function of GI classes found in this network. We also discuss how these GI classes contribute to the genomic robustness and the adaptive evolution of multicellular organisms.

## Introduction

The behaviour of biological systems and their adaptation to environmental changes depend on many factors on the path from genomic structure, through gene expression, molecular and functional interactions, to phenotypic manifestations. To simplify studies of these different levels of information, systems biologists may build a theoretical framework where biological systems are decomposed into six abstraction levels [1]: the genome structure (level I), the gene expression (level II), the physical interaction between systems elements (protein, DNA, RNA, etc. level III), the functional relationship between these elements (level IV), their biological and molecular function (level V) and the phenotypical manifestations (level VI). Within this framework, genetic interactions (GIs) are located at the level IV together with signaling and metabolic pathways [1].

The identification of a GI between two genes reveals that a mutation on the first one alters the biological consequences (the phenotype) associated to a mutation on the second one. Mapping GIs represents an important approach in understanding the link between genotype and phenotype. It is also a critical step to understand the robustness of biological systems–i.e. how the system compensates for the alteration of a function. Mapping GIs in human also recently emerged as a necessity to identify biomarkers from Genome-wide association studies (GWAS) and consequently, move the medical field towards a more personalized practice [2].

To date, only few (primarily unicellular) organisms have been amenable to experimental genome-wide screening approaches for mapping GIs. Thus, most of our information on the structure and the function of GI networks has been restricted to yeast (reviewed in [1] and [3]). Extensive studies on GIs in these systems showed clear relationships between GI networks and networks located at other abstraction levels. These studies revealed the relationship of GIs with signaling and metabolic pathways [4–7], between co-expressed genes, and between genes coding for interacting proteins [8,9]. They also identified the relationship between GIs, bioprocesses and phenotypes [10–12]. They characterized the degree of connectivity of genes within GI networks and assessed their enrichment in genes with high connectivity (GI-Hubs) as well as in multifunctional and essential genes [4,6,10,13]. Importantly, these studies identified dense subnetworks within the GI network (GDS) [10]. They showed that GDS tend to lay between molecular machines, that we will define in this study as dense subnetworks of protein-protein and protein-DNA interactions [6,8,9]. They also showed that GDS are monochromatic, i.e. they are composed of either positive (suppressive/alleviating) or negative (synergistic/aggravating) GIs [14–17], and are functionally biased [6,18]. These studies connect four abstractions levels (levels II to V), showing that GI networks coordinate molecular machines within bioprocesses.

These studies using yeast as a model brought also precious information on the role played by GIs in genomic robustness and evolutionary processes [7,19,20]. For example, these studies identified two separate groups of duplicated genes within distinct GDS: a group composed of redundant genes playing an important role on genomic robustness of systems and a group of redundant genes with divergent biological functions and with expected reduced impact on robustness [7]. In addition, they revealed that positioning of GIs within or between PPI-dense subnetworks (PDS) had an impact on their evolutionary conservation: GIs within PDS being more conserved than GIs between PDS [19,20].

To date, the structure and the function of GI networks are still largely unknown in multicellular organisms. Characterizing these networks is therefore required to better understand the genomic robustness of these systems and also how functional relationships between alleles influence phenotypical outcomes and evolutionary processes in multicellular contexts. To address this problem, we provide the first deep characterization of a network composed of ~600 GIs in a multicellular organism, the nematode *Caenorhabditis elegans*. This study aims to identify functional properties associated with GIs and groups of GIs and to understand better how the structural and functional organization of a GI network links molecular machines (abstraction level III) to bioprocesses, phenotypes and diseases (abstraction levels V and VI) in a multicellular context. Our results indicate that GIs form a heterogeneous group of entities when considering biological data located at different abstraction levels in *C*. *elegans*. We describe the specific characteristics of GI classes with respect to the connectivity degree within the GI- and the PPI-networks, their relationship with protein-protein interaction dense subnetworks (PDS), signaling and metabolic pathways and with phenotypic manifestations (essentiality, pleiotropy). We also discuss the impact of this structure on *C*. *elegans* genome robustness and evolution.

## Results

### Defining six classes of genetic interactions

Considering that the function and the structure of genetic interaction networks are mainly unknown for multicellular organisms while being of increasing interest, we characterized a network composed of ~1,500 GIs of the nematode *C*. *elegans*. To do so, we first investigated whether GIs constitute a heterogeneous group of entities in this organism and consequently, whether we could identify several GI classes with distinctive biological properties in this network.

We retrieved 1,514 genetic interactions (GIs) from Wormbase, Biogrid and the literature as described in the Methods section and in S1 Table. This set of GIs, called GIs-all, is composed of 750 (49.5%) interactions identified as experimentally validated GIs by either Wormbase and Biogrid curation systems (see Methods) or manually curated from the literature in our laboratory [21]. The remaining 764 GIs (50.46%) were identified using Textpresso, an automatic text mining system [22]. To test the false-positive rate in this later GI set, we manually curated 261 of these interactions and found that 252 of them (96.55%) were true-positives (experimentally validated GIs; S1 Table). Overall, GIs-all is predicted to contain at least 98% of validated GIs.

**Fig 1:**
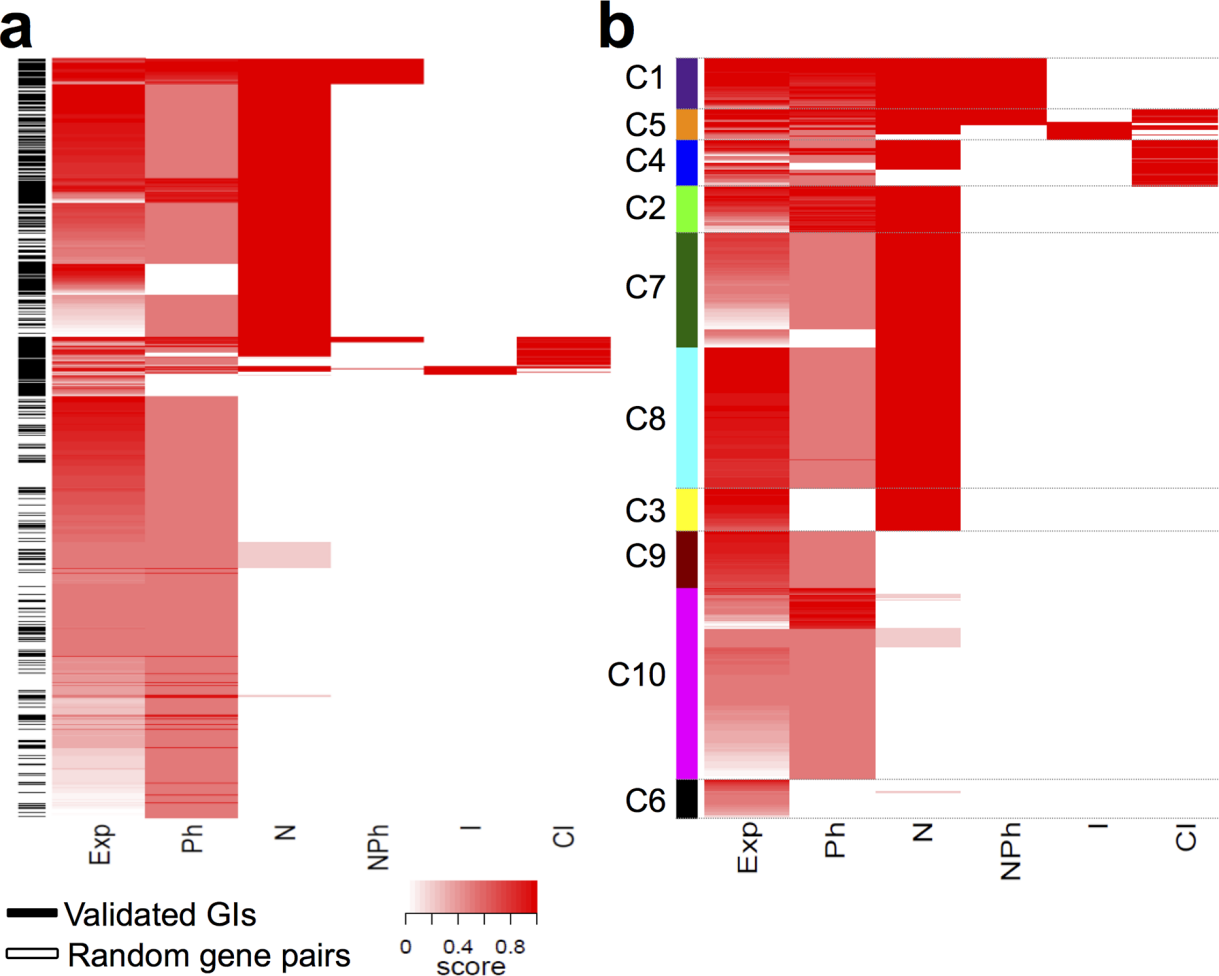
Identifying GI classes. **(a)** Positive (GIs, black lines) and negative (Random gene pairs, white lines) examples of genetic interactions were clustered based on their attribute scores using unsupervised methods. Columns show values for the six attributes used to predict interactions in [54]. Each individual attribute is either a measure of genes co-expression levels (*Exp*) or enrichment of shared phenotypes (*Ph*). They are also indicator for whether the neighborhoods of the genes of interest are enriched with the same phenotype (*N*). Here we define the neighborhood of a given gene as the set of genes that show significant co-expression with it (*P* ≤ 0.05, see Methods) and/or encode proteins that exhibit a PPI with the product of this gene. *NPh* is an indicator like *N* with the additional requirement that the genes of interest themselves must also exhibit the phenotype enriched in their neighborhoods. Attributes *I and CI* indicate that interacting genes code for interacting proteins or for proteins sharing a significantly high number of common protein-protein interaction partners. Scores correspond to the following valuation: 1 or 0 (on a binary system) for *N*, *NPh* and *I* attributes. (1 –(*P*-value <0.05)) for *Exp*, *Ph* and *CI* attributes. **(b)** Positive examples of genetic interactions were clustered and identified by the color code on the left.

Statistical attributes using expression, protein-protein interaction (PPI) and phenotypic data were previously described as powerful tools to segregate GIs-all from a set of gene-pairs randomly selected from the genome [21]. GIs being shown as rare events [10], we expect the latter set of gene-pairs to be mostly composed of “true” negative examples of GIs (see Methods). Attributes used in this study capture the level of co-expression between interacting genes (Exp, Fig 1A and 1B), the enrichment of shared phenotypes (Ph, Fig 1A and 1B), their ability to encode proteins that interact physically (I, Fig 1A and 1B) and/or have more common interacting-partners than expected by chance alone (CI, Fig 1A and 1B). Two attributes were also designed to identify GIs within functional modules (N and NPh, Fig 1A and 1B). The attribute called "Neighborhood" (N) identifies the enrichment of phenotypes in the neighborhoods of gene-pairs within a network where genes are linked when they are significantly co-expressed, or code for interacting proteins (see Methods for details; [21]). The attribute "Neighborhood with Phenotype" (NPh) identifies pairs of genes with a positive value for the attribute N and the association of both genes with the phenotype enriched in their neighborhood (see Methods for details; [21]). We used these attributes to assess whether GIs display distinctive dependency towards data located at different abstraction levels. To do so, attribute values were used to cluster GIs-all together with 1,500 gene-pairs randomly chosen in the *C*. *elegans* genome. If GIs were forming a homogeneous group of entities with unique distinctive properties when considering negative sets of examples, we would expect them to cluster as a group from these negative examples. However, GIs-all tend to cluster into groups of GIs (using Euclidean distance-based dissimilarities; see Methods) with some dispersion among the negative examples (Fig 1A). This suggests that GIs-all can be subdivided into GIs groups with potential distinctive biological properties.

In an attempt to characterize the function of these GI groups, we used a cluster selection algorithm to identify GI classes from GIs-all (Fig 1B), while controlling the robustness of this classification (see S1 Text and S1 Fig). This study identified ten GI classes, six of them (C1-C6) being significantly different from gene-pairs randomly selected from the genome (see S1 Text). Analysis of the proportion of missing data supporting each class revealed that missing expression, phenotype and/or PPI data could not explain the classification by itself. We conclude from this analysis that GI classes with similar combination of attribute values would still be identified from a GI set exempt of missing data S2 Fig.

### GI classes have different functions

Two main classes of GIs have been identified in the yeast genome: positive (alleviating interactions) and negative (synergistic interactions) [8,12,23–25]. Monochromaticity of GI dense subnetworks (GDS), enriched in either positive or negative interactions, has also been identified in yeast [7,18]. These monochromatic GDS were described as being functionally biased—i.e. they tend to be associated with specific biological functions [7,18]. This characterization of GI networks in yeast was critical to understand better the role of GIs in coordinating gene function within biological processes.

To characterize the potential distinctive biological properties of GI classes and their ability to control different bioprocesses, we first assessed whether GIs tend to form dense subnetworks (GDS) in *C*. *elegans*. We also tested whether these GDS may be monochromatic or multichromatic—if they were enriched in one or several GI classes. Lastly, we assessed whether these GI classes are functionally biased.

This analysis revealed that GIs from GIs-all form few GDS (see S1 Text and S3A Fig). However, while the number of GDS formed is too small to assess their enrichment in unique class or combination of classes as done in yeast, clustering of classes based upon the GDS composition, revealed that GI classes tend to form GDS in a biased manner: C1 with C2, C4 with C5 and C3 with C6 (see S1 Text and S3A Fig). Analysis of the gene composition of GI classes also revealed that C4 GIs share more genes with C5 than with the other classes (see S1 Text and S4 Fig) supporting the hypothesis that GI classes may assemble into GDS in a biased way.

**Fig 2:**
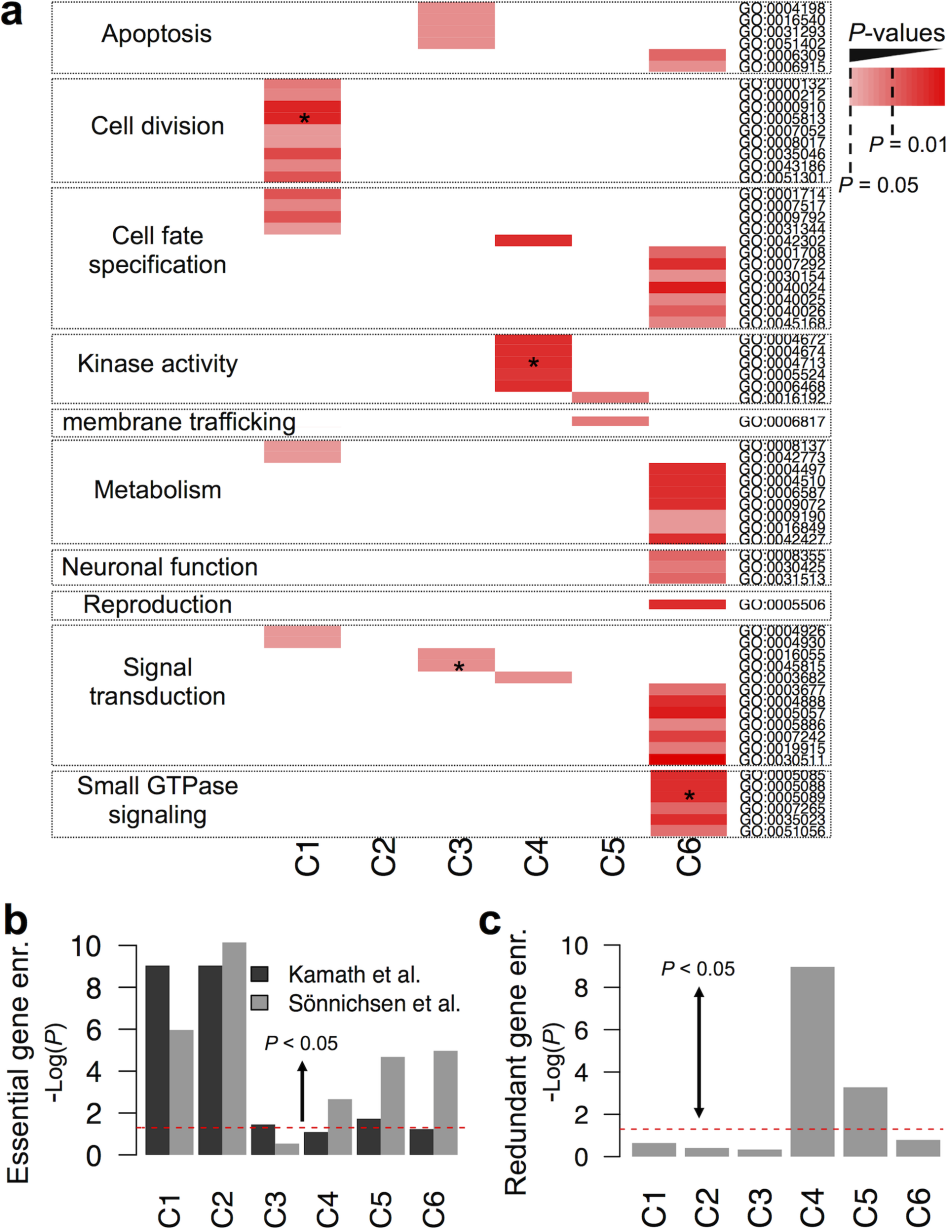
GI classes have different functions. **(a)** Summary of Gene Ontology (GO) term enrichments for interacting genes in individual genetic interactions (GIs) classes when compared to interacting genes from GIs-all. Only the statistically significant enrichments are shown with adjusted *P*-values. An asterisk (*) means that the GI class is also enriched with the functional annotation when gene repetitions were not considered (S2 Table). **(b)** Enrichments of essential gene sets (Kamath et al. and Soninchsen et al. taken from [28] and [29]) in GI classes when compared to GIs-all. **(c)** Enrichment of redundant and evolutionarily conserved genes in GI classes when compared to GIs-all. The redundant gene list was made of 306 genes taken from [30]. (b-c) -Log_10_ of *P-*values obtained using Fisher’s exact tests are indicated. The area over the red dashed line indicates significant enrichment (*P* < 0.05).

Enrichment of GI classes in Gene Ontology annotations (GO, [26]) was also measured. GO annotations were found enriched for all GI classes except C2 (Fig 2A and S2 Table and S5–S11 Figs). This analysis was done first by considering gene repetition within classes (some genes being involved in more than one GI in a given class) and secondly, without considering gene repetition (asterisk indicates enrichment observed without considering gene repetitions; Fig 2A and S2 Table). Similar results were obtained for both analyses, suggesting that GI classes are functionally biased as detailed below.

**Fig 3:**
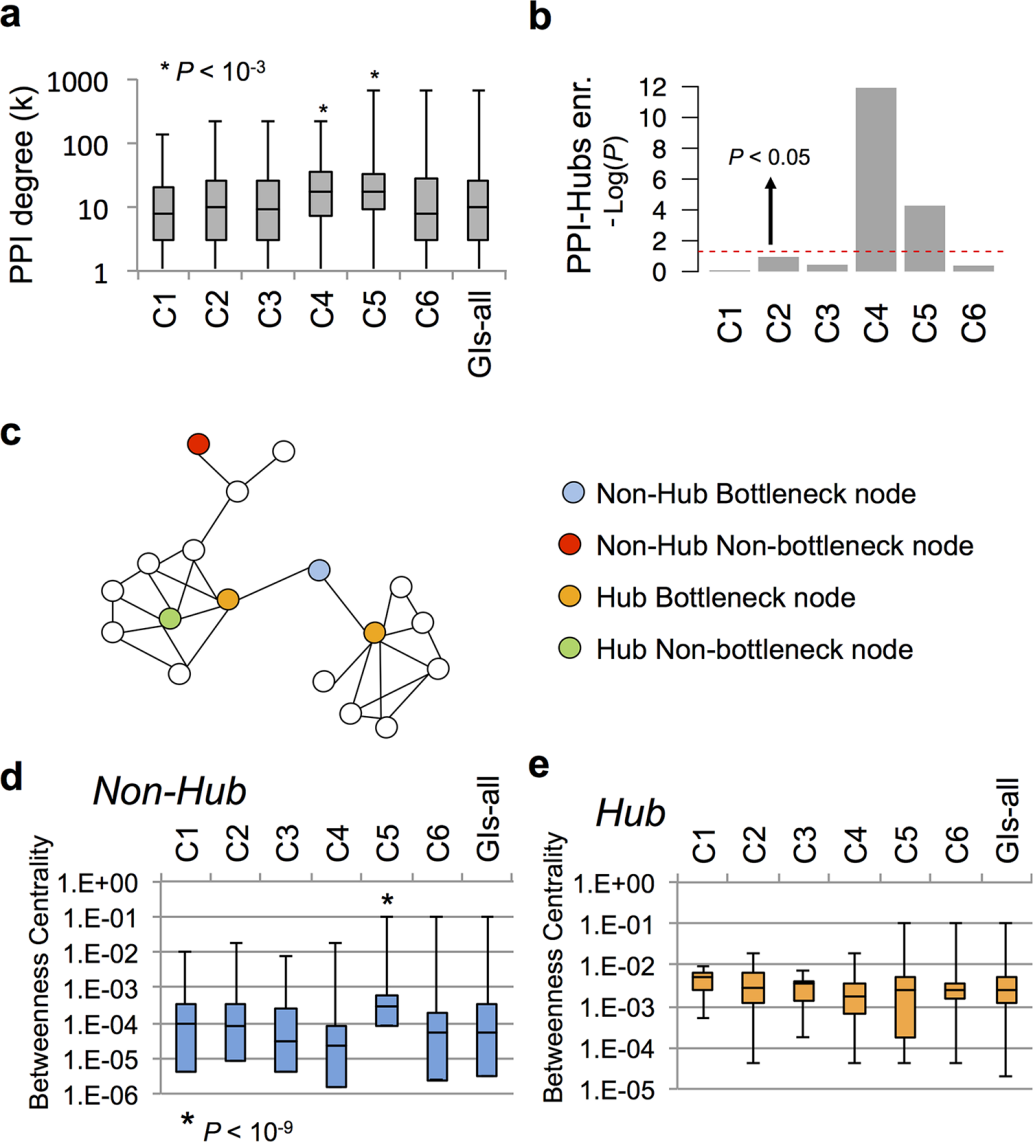
GI classes have different position in PPI-network. **(a)** Physical interaction degree of gene products in the protein-protein interaction (PPI) network (only genes coding for proteins with at least one interacting partner were considered). *P < 10^−3^, Wilcoxon rank-sum test, indicates significant difference when compared to GIs-all and other GI classes. **(b)** Enrichment of PPI-Hubs in GI classes when compared to GIs-all. -Log_10_ of *P-values* obtained using Fisher’s tests are indicated. The area over the red dashed line indicates significant enrichment (*P* < 0.05). **(c)** Illustration of bottlenecks and the different type of nodes in the protein-protein interaction (PPI) network (adapted from [36]). **(d-e)** Betweenness centrality of non-Hub and Hub gene products. *P < 10^−3^, Wilcoxon rank-sum test, indicates significant difference when compared to other GI classes. The box plots (a, d-e) represent the min, max, 25^th^, 50^th^ (median) and 75^th^ percentile of either PPI Degree (k) or betweenness centrality values.

C1 interactions were enriched in genes involved in cell division (*P* = 0.01, Fisher’s exact test; Fig 2A and S2 Table). For example, C1 GIs were identified between *spd-2*, *spd-5* and *dhc-1* controlling centrosome assembly and maturation [27] (S5 Fig). We expect genes involved in cell division to be essential. To test a potential enrichment of essential genes in C1, we measured the enrichment of two gene sets of 294 and 1,259 essential genes identified using systematic RNA silencing by Kamath *et al*. [28] and Sonnichsen *et al*. [29] in GI classes when compared to GIs-all using a hypergeometric test (bars above the red line indicate a significant enrichment; *P* < 0.05; Fig 2B). These data showed that C1 and C2 display the highest enrichment level in essential genes when compared to GIs-all and other classes. These data support the functional bias of C1 towards cell division and also associate GDS enriched in C1 and C2 interactions to essential biological functions. Enrichment analysis of GO terms within GI classes also revealed that C4 and C5 were enriched in genes coding for kinases (*P* < 0.0001, Fisher’s exact test; Fig 2A and S2 Table). A kinase-centric study of the GI network in yeast showed that genes coding for kinases are often redundant and are involved in either kinase-kinase (K-K) or kinase-substrate (K-S) GIs [18]. We assessed whether GI classes were enriched in these two kinds of interactions when compared to GIs-all. In this study, kinases were identified based on their GO annotation (S2 Table) and substrates were identified as non-kinases. This analysis revealed that C4 is significantly enriched in K-K (*P* = 0.004, Fisher’s exact test) and K-S (*P* = 0.03, Fisher’s exact test). Considering that K-K interactions were primarily observed between redundant kinases [18], we assessed whether C4 was also enriched in GIs between redundant genes. We thus measured the enrichment in GI classes of 306 genes identified as redundant (linked by a synthetic sick or lethal interaction) and evolutionarily conserved genes [30]. This analysis revealed that both C4 and C5 were enriched in evolutionarily conserved redundant genes (Bars above the red line indicate significant enrichments; *P* < 0.05; Fig 2C). In order to assess whether C4 and C5 were enriched in interactions involving only redundant kinases or redundant genes at large, we measured this enrichment after removing kinases from both the Tischler *et al*. list of conserved redundant genes and from GI classes. This analysis revealed that only C4 was enriched in GIs involving non-kinase redundant genes (Fisher’s exact test; *P* = 9 x 10^−6^). This suggests that C5 GIs involve mainly conserved redundant kinases while C4 GIs involve conserved redundant genes coding for kinases or not. Examples of C4 GIs between conserved genes coding for redundant kinases and non-kinases are the interactions between the type I and type II TGF-beta receptor coding genes *daf-1* and *daf-4* [31–33]; and between the three Rho GTPases *ced-10*, *mig-2* and *rac-2* shown to control cell migration [34] (S5 Fig). Moreover, GO annotation enrichments revealed that C3 was enriched in genes involved in cell signaling (*P* = 0.01, Fisher’s exact test; Fig 3A) and C6 in small GTPase signaling (*P* = 0.001, Fisher’s exact test; Fig 3A).

Altogether, these data suggest that GI classes may assemble in GDS in a “class-biased” manner and also display a functional bias. Genes interacting through C1 and C2 GIs tend to be essential genes, more particularly involved in cell division. Genes interacting through C4 or C5 GIs tend to code for evolutionarily conserved redundant kinases and non-kinases. This study also suggests a potential function for genes interacting through C3 and C6 GIs in cell signaling.

### GI classes have different positions in the PPI network

The coordination of molecular machines by GIs has been intensively investigated in yeast through characterization of the relationship existing between GIs and protein-protein interaction (PPI) networks [5,8,9]. Four out of the six attributes used in this study are built, even partially, on PPI data (N, NPh, CI, I; see Methods). GI classes, displaying positive values for different combinations of attributes (Fig 1B), may exhibit distinct relationship with the PPI network. To assess the relationship between GI classes and molecular machines we measured the average PPI-degree of proteins coded by genes within GI classes and GIs-all (Fig 3A). This revealed that C4 and C5 GIs involve genes with a median PPI-degree significantly higher than the other classes and GIs-all (*P* < 10^−3^ and *P* < 10^−6^ respectively, Wilcoxon rank-sum test; Fig 3A). We then assessed whether this was due to a significant enrichment of genes coding for PPI-Hubs—defined as the 20% proteins with the highest PPI-degree within the PPI network (Fig 3B and S12 Fig). This revealed that C4 and C5 were indeed significantly enriched in PPI-Hubs when compared to GIs-all (*P* < 10^−4^ and *P* < 10^−11^ respectively, Fisher’s exact test; Fig 3B).

The structure of the PPI networks has been extensively studied in unicellular and multicellular organisms [35]. As shown for GIs, PPI tend to assemble into PPI-dense subnetworks (PDS) orchestrated around nodes. These nodes can either be Hubs or non-Hubs, with distinct structural functions within the PPI network [35,36]. The betweenness centrality metric was previously defined to identify network nodes playing a critical role in the PPI network organization (called “bottleneck nodes”) [36]. These nodes were characterized by their positioning in many shortest paths within the PPI network ([36], Fig 3C). Measurement of the betweenness centrality of PPI-non-Hubs and PPI-Hubs in the different GI classes revealed that C5 non-Hub proteins tend to be bottlenecks with a significantly higher betweenness centrality than GIs-all (*P* < 10^−9^, Wilcoxon rank-sum test; Fig 3D). The betweenness centrality of PPI-Hubs was not significantly different between GI classes and GIs-all (Fig 3E).

Altogether, these data clearly show that GI classes have distinctive properties with respect to the PPI network and may consequently coordinate differently molecular machines in biological processes. They identify C4 and C5 interactions as enriched in genes coding for PPI-Hubs that tend to be non-bottlenecks (green node; Fig 3C), suggesting that these Hubs may be embedded within PDS rather than at their periphery. C5 also appear to be enriched in non-Hub Bottleneck nodes (Blue nodes, Fig 3C) thought to play a critical role in PDS coordination [36]. Therefore, these data suggest, C4 and C5 interactions may play a critical role in controlling the assembly and the coordination of PDS.

### GI classes are either PDS-centric or PDS-independent

**Fig 4:**
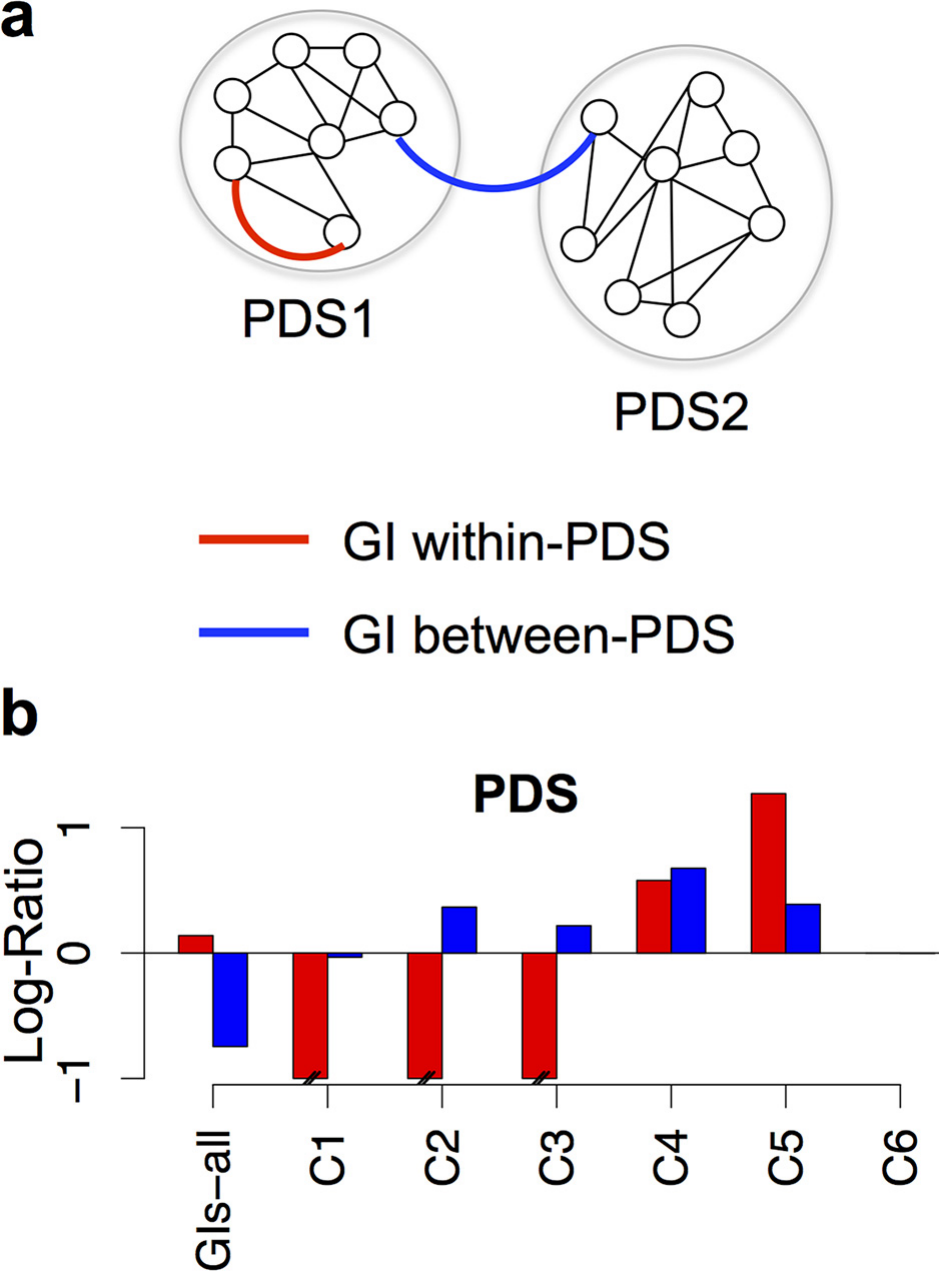
GI classes define PDS-depend and -independent modules. **(a)** Schematic representation of possible relationships between genetic interactions (GIs) and protein-protein dense subnetworks (PDS). Each node represents a gene being part of PDS1 or PDS2. A red line indicates a within-PDS GI and a blue line, a between-PDS GI. **(b)** Log-Ratio scores for within-PDS (red bars) and between-PDS (blue bars) relationships observed between genes interacting through different GI classes, or present in GIs-all. A positive Log-Ratio score means that the frequency of within- or between-PDS GIs occurring in the GI class is significantly higher than the frequency of similar situations witnessed in relevant randomized GI networks with a probability of 0.99 (see Methods).

The data presented above suggest that C4 and C5 GIs link genes coding for proteins within or between PDS while C1, C2, C3 and C6 may link genes coding for proteins outside PDS. To test this hypothesis we used the Cytoscape “MINE” plugin [37] to identify PDS within the PPI-network as previously done [8,9] (see Methods). This approach identified 106 PDS containing at least four proteins. We then assessed whether GIs-all and GI classes were enriched in genes coding for proteins: (i) in the same PDS (GI within-PDS; red lines / bars; Fig 4A and 4B); or (ii) between PDS (pairs of proteins located in different PDS; GI between-PDS; blue lines / bars; Fig 4A and 4B). To evaluate this enrichment level, we compared the frequencies obtained for the different scenarios (within-PDS and between-PDS) with those for randomized GI networks of similar size and structure, and computed log-ratio transform (LR) scores (see Methods, Fig 4B). Consistently with previous studies [38], GIs-all was enriched in within-PDS connections while being depleted in between-PDS connections (Fig 4B), confirming that GIs in *C*. *elegans* are more frequently observed within-PDS than between-PDS. Interestingly, C2 and C3 GIs tended to occur between-PDS, and C4 and C5 GIs occur both within- and between-PDS, while GIs in C1 and C6 appear to be independent from PDS (Fig 4B). We assessed whether this PDS-independency results from a dependency of C1 and C6 towards connected 3-protein triangles, called bistable motifs, which are not considered as PDS in our study (see Methods). However, no bistable motif was found in GIs-all suggesting that C1 and C6 GIs are independent from PDS.

These data confirm our hypothesis that C4 and C5 GIs are enriched in within- and between-PDS. We will consequently, characterize them as PDS-centric interactions, and C1 and C6 GIs as PDS-independent. Moreover, C2 and C3 GIs appeared to be enriched in between-PDS connections suggesting some kind of functional relationship with the PDS-centric interactions. Taken together these data suggest that GI classes display distinct relationships towards PDS and consequently, may coordinate differently molecular machines within biological processes.

### PDS and pathways constitute different functional modules

In the yeast *S*. *cerevisiae*, subgroups of GIs have been identified and characterized based on their relationship with physical interaction networks–mainly protein-protein (PPI) and protein-DNA (PDI) interactions [5,8,9]. In these studies, a " pathway " was defined as a dense subnetwork or a connected graph module of PPIs (defined as PDS in our study) and PDIs. These studies showed that GIs tend to occur rather between- than within-pathways [5,8,9]. Interestingly, a similar study was done on GI networks in *C*. *elegan*s and showed the opposite–GIs occur more frequently within- than between-pathways [38,39]. These results are in agreement with data obtained in this study for GIs-all (Fig 4B).

**Fig 5:**
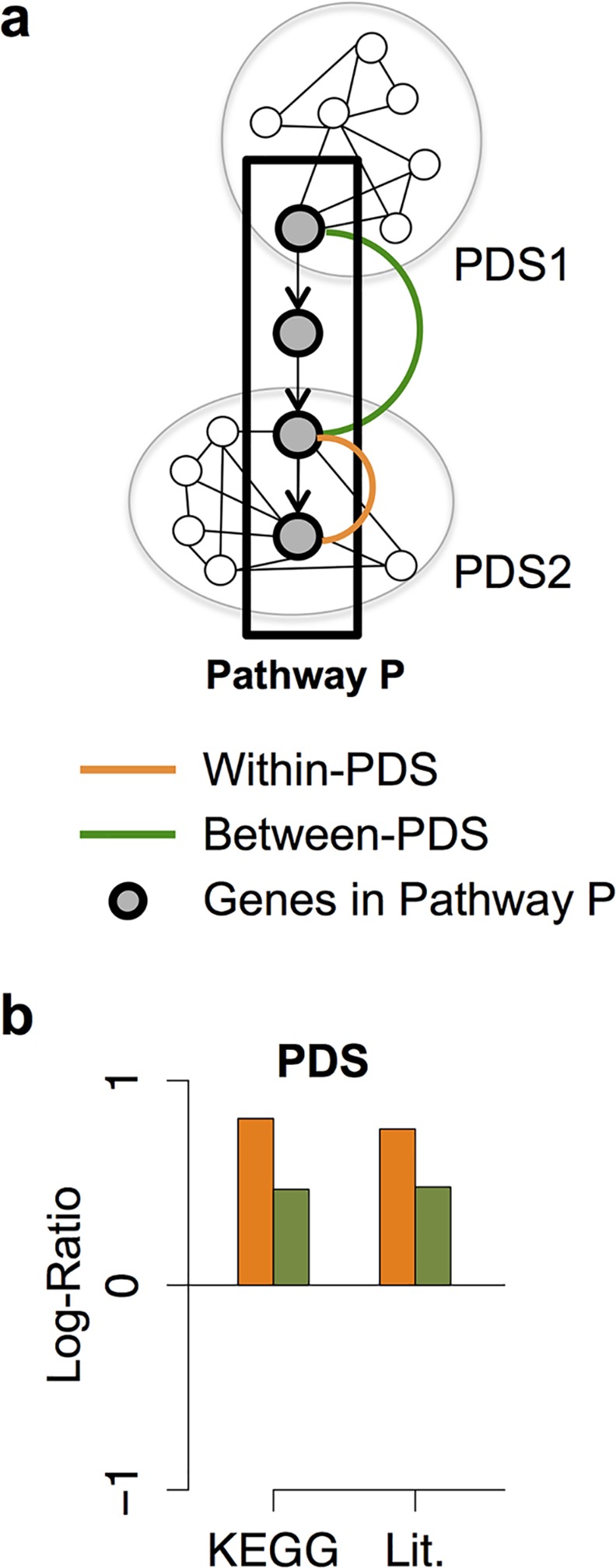
PDS and pathways are different functional modules. **(a)** Schematic representation of possible relationships between Pathways and protein-protein interaction dense subnetwork (PDS). Each node represents a gene product connected by a PPI (black lines) or by a functional relationship within a pathway P (back arrows). The grey nodes represent gene products involved in a pathway P. An orange line linking two grey nodes indicates that these two genes/proteins interact within-pathway and also within-PDS. A green line linking two grey nodes indicates that these two genes/proteins interact within-pathway and between-PDS. **(b)** Log-Ratio scores for within-PDS and between-PDS relationships occurring within-pathways. A positive Log-Ratio score means that the frequency of within-pathway relationships occurring also within-PDS (orange bars) or within-pathway relationships occurring between-PDS (green bars) is higher than the frequency of similar situations witnessed in relevant randomized pathway networks.

The pathway described above is quite different from the definition used by developmental geneticists for whom a pathway consists in a group of genes functioning together to control a given biological process [40]. Characterization of the relationship between PDS and pathways is consequently required to clarify the relative positioning of GIs and pathways (both located at the abstraction level IV) between molecular machines (level III) and biological processes (level V). To do so, 61 pathways were retrieved from the KEGG database [41] along with 33 pathways controlling the embryonic and larval development of *C*. *elegans* that we manually curated from the literature (S3 Table; see Methods). We then assessed whether gene-pairs involved in a given pathway (within-pathway interactions) tend to code for proteins, which are part of the same PDS (within-PDS; orange lines / bars; Fig 5A and 5B) or of two distinct PDSs (between-PDS; green lines / bars; Fig 5A and 5B). We compared the frequencies obtained for these scenarios with those resulting from randomization of pathways (see Methods, Fig 5B). Interestingly, proteins coded by gene-pairs in the same KEGG or pathways from the literature (KEGG and Lit. respectively; Fig 5B) are enriched in within- and between-PDS interactions (orange and green bars respectively; Fig 5B). We also confirmed that this enrichment of within- and between-PDS observed within-pathways did not depend on the topology of the PDS network but instead depend upon the interactions themselves (see S1 Text and S14 Fig).

These data suggest that pathways and PDSs are distinct functional modules. They also suggest that pathways are composed of several PDS and that proteins involved in a given pathway may be part of the same PDS or of different PDSs. We confirmed these assumptions through a close examination of genes/proteins involved in pathways and PDSs as shown in S4 Table and detailed in supplementary information (S1 Text).

### GI classes are enriched in within-pathway interactions

**Fig 6:**
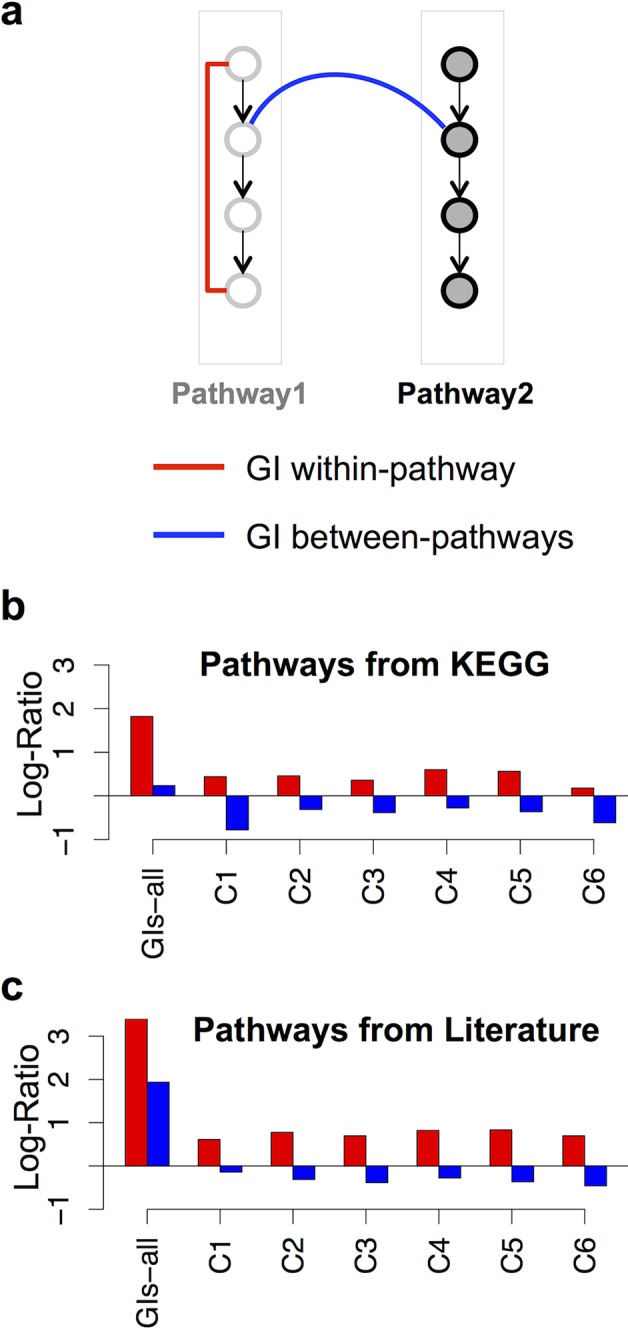
GI classes are enriched in within-pathways interactions. **(a)** Schematic representation of possible relationships between genetic interactions (GIs) and pathways. Each node represents a gene being part of pathways 1 or 2. A red line indicates a within-pathway GI and a blue line, a between-pathway GI. **(b-c)** Log-Ratio scores for within-pathway (red bars) and between-pathway (blue bars) relationships observed between genes interacting through different GI classes, or present in GIs-all. A positive Log-Ratio score means that the frequency of within- or between-pathways GIs occurring in the GI class is significantly higher than the frequency of similar situations witnessed in relevant randomized GI networks with a probability of 0.99 (see Methods).

Considering that pathways and PDS are distinct functional modules and that GI classes have distinct relationships towards PDS, we were interested to test whether this would also be the case for pathways. We thus investigated if GI classes and GIs-all were enriched in within-pathways (pairs of genes functioning in at least one common pathway, see Methods) and/or between-pathways (pairs of genes involved in at least one pathway but not involved in any common pathway, see Methods; Fig 6A). As detailed for PDS in the previous sections, we compared the frequencies obtained for the different scenarios (within-pathways and between-pathways) with those for randomized GI networks (Fig 6B and 6C, see Methods). GI classes and GIs-all were enriched in within-pathway interactions for both pathways retrieved from the KEGG database and from the literature (Fig 6B and 6C respectively). Remarkably, GI classes were depleted in between-pathway interactions while GIs-all was enriched in this kind of interactions (Fig 6B and 6C). These results suggest that non-selected clusters, C7 to C10 (Fig 1B), are enriched in between-pathway interactions. Characterization of these later GI classes revealed that it was indeed the case: except for C10 that was enriched only in within-pathway interactions, C7, C8 and C9 GIs were enriched in both within- and between-pathways interactions (S13 Fig).

Together, these data show that C1/C6 PDS-independent, C4/C5 PDS-centric and C2, C3 GIs are enriched in within-pathways interactions. This implies that pathways involve both functional interactions that are organized around PDS and other that are independent from PDS.

### C3 and C6 GIs involve connectors

**Fig 7:**
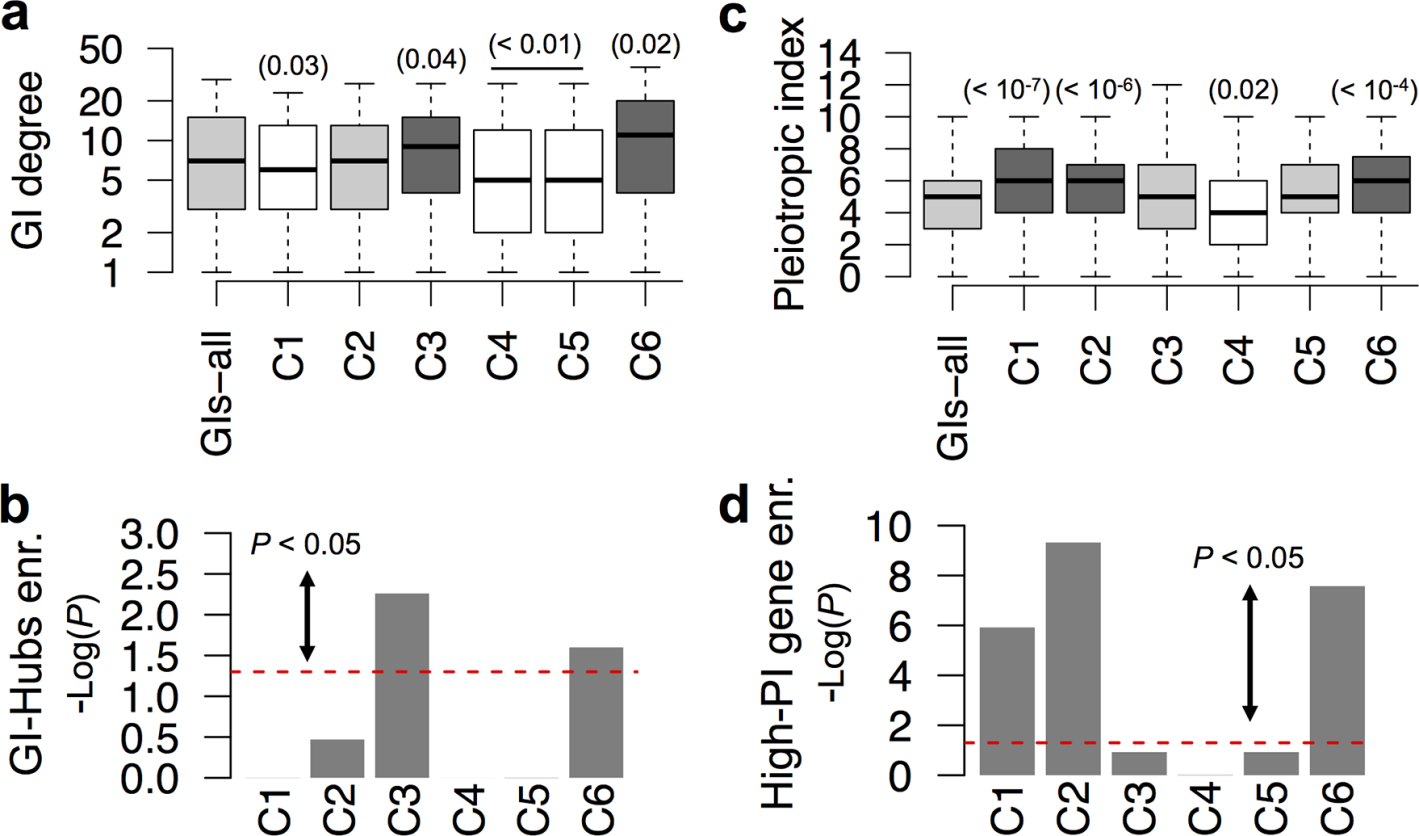
Defining pleiotropic and non-pleiotropic connectors. **(a)** Box plots showing distributions of genetic interaction (GI) degrees for GIs-all and the six classes of GIs. Distributions were found to be significantly higher (Dark grey boxes) or lower (white boxes) than GIs-all (*P* < 0.05, Wilcoxon rank-sum test). Light grey boxes indicate classes with GI degree not significantly different than GIs-all. **(b)** Enrichment in GI-Hubs (defined as the 20% most connected genes in GIs-all) for GI classes when compared to GIs-all. **(c)** Distribution of pleiotropic indices (PIs) for GIs-all and the six classes of GIs. Dark grey, white and light grey boxes are designated as in panel (a). **(d)** Enrichment in High-PI (defined as the top 20% of genes with the highest PIs genome-wide) for GI classes when compared to GIs-all. The box plots (a, c) represent the min, max, 25^th^, 50^th^ (median) and 75^th^ percentile of either GI Degree (a) or pleiotropic indices (c). (b, d) -Log_10_ of *P*-values obtained using Fisher’s tests are indicated. The area over the red dashed line indicates significant enrichment (*P* < 0.05).

Highly connected genes called connectors, or GI-Hubs, are genes whose alteration impacts on a large number of genes. Their function is consequently expected to be central within pathways and bioprocesses [5,6,39]. GI-Hubs are defined here as the 20% genes with the highest GI degree within the GI-network (S16A Fig). In order to understand better the function of GI classes within pathways, we characterized the distribution of GI-Hubs/connectors within GIs-all and their potential enrichment in GI classes. Therefore, we computed the average GI degree of interacting genes in GI classes and GIs-all (Fig 7A), and also measured their enrichment in GI-Hubs (Fig 7B). This analysis revealed that C3 and C6 showed a median GI-degree significantly higher than GIs-all and are enriched in GI-Hubs (Fig 7A and 7B). These results suggest C3 and C6 are enriched in connectors that may play a critical role in the coordination of gene functions within pathways. Such a function for C3 and C6 GIs is consistent with their expected involvement in cell signaling (Fig 2A).

### Identifying pleiotropic and non-pleiotropic GIs

Our study supports a model in which four out of the six classes of GIs coordinate the function of genes either in a PDS-centric or in a PDS-independent manner. They also identify two additional classes of GIs with central coordination function through the involvement of connectors/GI-Hubs. *C*. *elegans* being a multicellular organism, one may ask whether some parts of this organization may be organ or process specific while others may be ubiquitous. To answer this question, we assessed whether GI classes are enriched in pleiotropic genes–i.e. genes whose genetic alteration is associated to multiple phenotypic expressions and consequently, whose function is required in several organs and/or at different developmental stages of the animal. To do so, we divided the *C*. *eleg*ans phenotype ontology into 22 groups of phenotypes (S15 Fig). We computed a pleiotropic index (PI) for each gene as the number of phenotype groups containing at least one phenotype expressed upon genetic alteration of the gene of interest (see Methods). The PI median genome-wide (2 ± 1.48 MAD) is smaller than in GIs-all (5 ± 2.97 MAD; S16B Fig), and its distribution revealed that a large number of genes displayed a low PI while a small number of genes have a high PI (S16B Fig). Using this distribution of PI, we defined a class of highly pleiotropic genes (High-PI) as the 20% genes displaying the highest PI genome-wide (S16B Fig).

We measured the PI of genes involved in GI classes and found that only genes involved in C1, C2 and C6 GIs displayed significantly higher median PIs than GIs-all (*P* < 10^−4^, Wilcoxon rank-sum test; dark grey boxes; Fig 7C) and are significantly enriched in High-PI genes (*P* < 10^−5^; Fisher’s exact test; Fig 7D). We further characterized the distribution of GI classes at different PI ranges and the ability of these classes to link genes between ranges (see S1 Text and S17 Fig). This study revealed that while C5 connects genes only within an average PI range, other classes connect genes across PI ranges, especially C3 and C6 that tend to link genes within an average PI range to either genes with low (C3 class) or high PI (C6 class).

These data suggest that PDS-independent GIs tend to involve pleiotropic genes while PDS-centric GIs involve genes with an average to low pleiotropy. They also further characterized the properties of connectors involved in C3 and C6 GIs, linking genes within different pleiotropic ranges: C6 and C3 GIs link genes from an average to a high-pleiotropy and from a low to an average pleiotropy respectively. These data show that the organization of the characterized GI network within pathways is more complex when considering the multicellular nature of *C*. *elegans*. They imply that genetic mutations within a given pathway may either lead to pleiotropic or tissue/developmental stage-specific phenotypic manifestations. This also implies that such mutation may be compensated by a mutation of a genes involved in the same pathways but with pleiotropic or non-pleiotropic effect.

### Integration of different kind of data is required to identify GI classes

**Fig 8:**
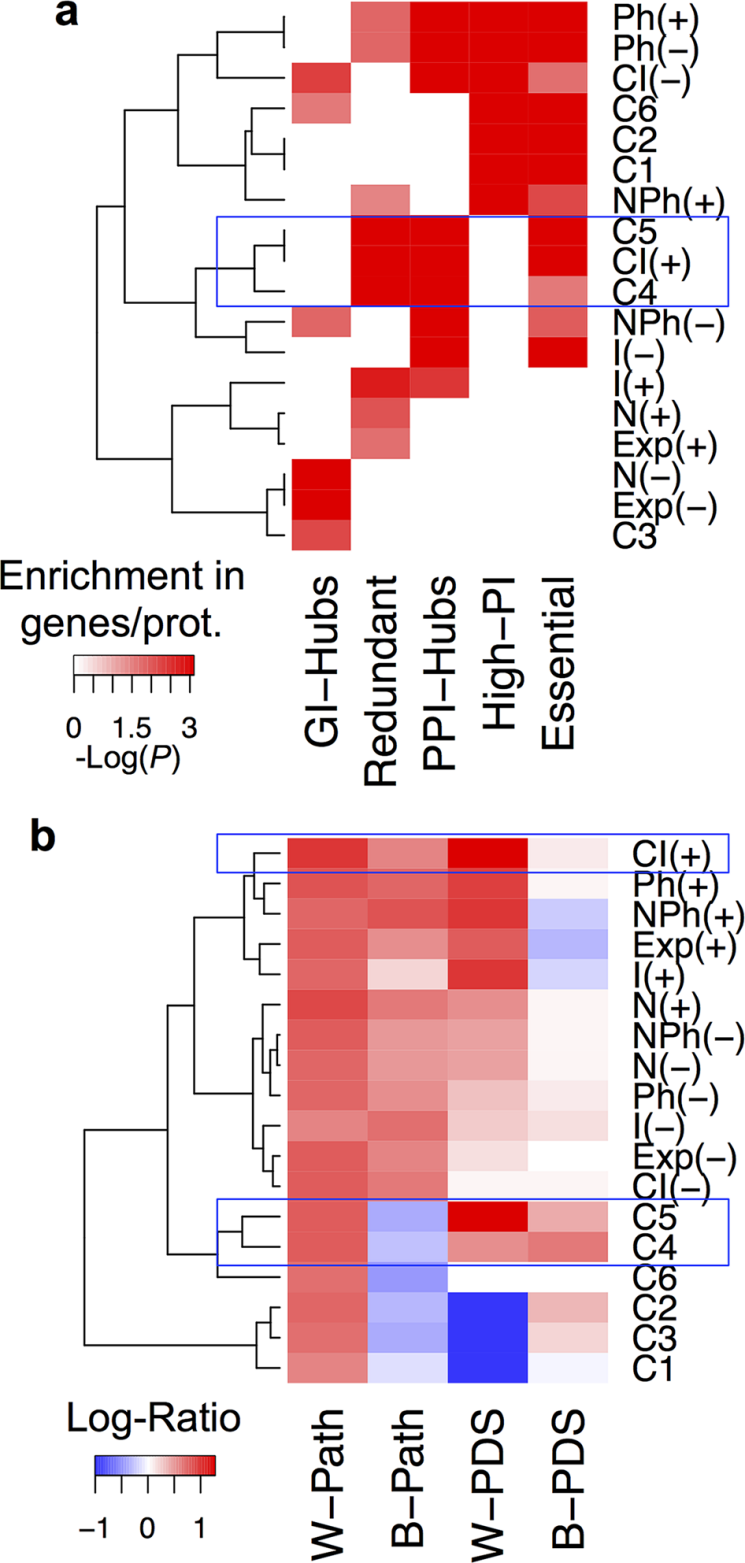
Data integration is required to identify GI classes. Hierarchical clustering of **(a)** Enrichment in GI classes and GI groups of Genetic interaction-Hubs (GI-Hubs), redundant genes, protein-protein interaction-Hubs (PPI-Hubs), Highly pleiotropic genes (High-PI) and essential genes -log of P-values from Fisher’s exact test are indicated. GI groups are associated to a positive value (+; above a threshold) or a negative value (-; below the threshold) for indicated attributes. Threshold values for each attributes are indicated S6 Table. **(b)** Log-Ratios profiles for GI classes and GI groups associated to a positive (+) or a negative (-) values for indicated attributes. Blue boxes indicate enrichment of biological characteristics for C4 and C5 GI classes and CI(+) GI group.

PDS-centric GIs (C4 and C5) are GI classes displaying positive values for I and CI attributes (Fig 1B), which are attributes mainly built on PPI network. We, consequently, wondered whether these two classes of GIs could have been identified considering PPI data alone. In order to test the value of the data integration strategy used in this study to identify the GI classes, we assessed whether functional characteristics observed for each class may depend on one or a combination of the attributes used for the classification (Fig 1A and 1B). To answer this question, we defined a threshold value for each attribute (S6 Table), allowing us to identify groups of GIs associated to a positive or a negative value per attribute. We subsequently assessed the enrichment in GI-Hubs, PPI-Hubs, redundant genes, essential genes, genes with high PI (High-PI; Figs 8A and S18), within- and between-PDS (W-PDS and B-PDS; Figs 8B and S19) as well as within- and between-pathway interactions (W-Path and B-Path respectively, Fig 8B) in these GI groups. We clustered GI groups and GI classes (C1-C6) based on these enrichment levels using Euclidean distances (Fig 8A and 8B). This analysis revealed that the enrichment of a given GI property is not associated to a positive or a negative value for a single attribute but for several of them (Fig 8A and 8B). For example, enrichment of genes with High-PI was observed in groups of GIs with positive or negative values for Ph (enrichment of phenotypic manifestations), with negative value for CI (high number of common partners within the PPI network), and positive value for NPh (enrichment of a phenotype associated to interacting genes in their respective neighborhoods). This suggests that integration of a set of attributes defines the biological functions of identified GIs.

This analysis also revealed that the biological characteristics observed for GI-classes are not found in GI groups associated to either a positive or a negative value for an attribute. For instance, when comparing C4 and C5 with the GI group positive for CI (interacting genes coding for proteins sharing a significantly high number of common PPI partners), these GI groups/classes displayed similar enrichment of redundant genes, PPI-Hubs and essential genes (blue square; Fig 8A) but have different enrichment profiles when considering their relative positioning towards pathways and PDS (blue square; Fig 8B). This suggests that the enrichment of essential genes, PPI-Hubs and redundant genes in C4 and C5 may be significantly influenced by a positive value for the CI attribute (Fig 1B). Positive value for this attribute does not, however, explain the depletion of C4 and C5 in GIs between-pathways and their enrichment in GI between-PDS (Fig 8B).

Altogether, this study demonstrates that the biological characteristics identified for GI classes depend on combination of attributes identified through data integration strategy. Importantly, it indicates which combination of attributes is appropriate in integration to identify classes of GI with specific biological functions.

## Discussion

Extensive mapping and characterization of the yeast genetic interaction network has revealed precious information on the still mysterious genetic interactome. These studies identified multiple classes of GIs with distinctive properties when considering the protein-protein interaction network and biological processes (bioprocesses) [5,7,8]. Therefore, these studies suggested that GIs display a certain level of heterogeneity. Whether such heterogeneity could also be observed in higher organisms was however still unknown.

**Fig 9:**
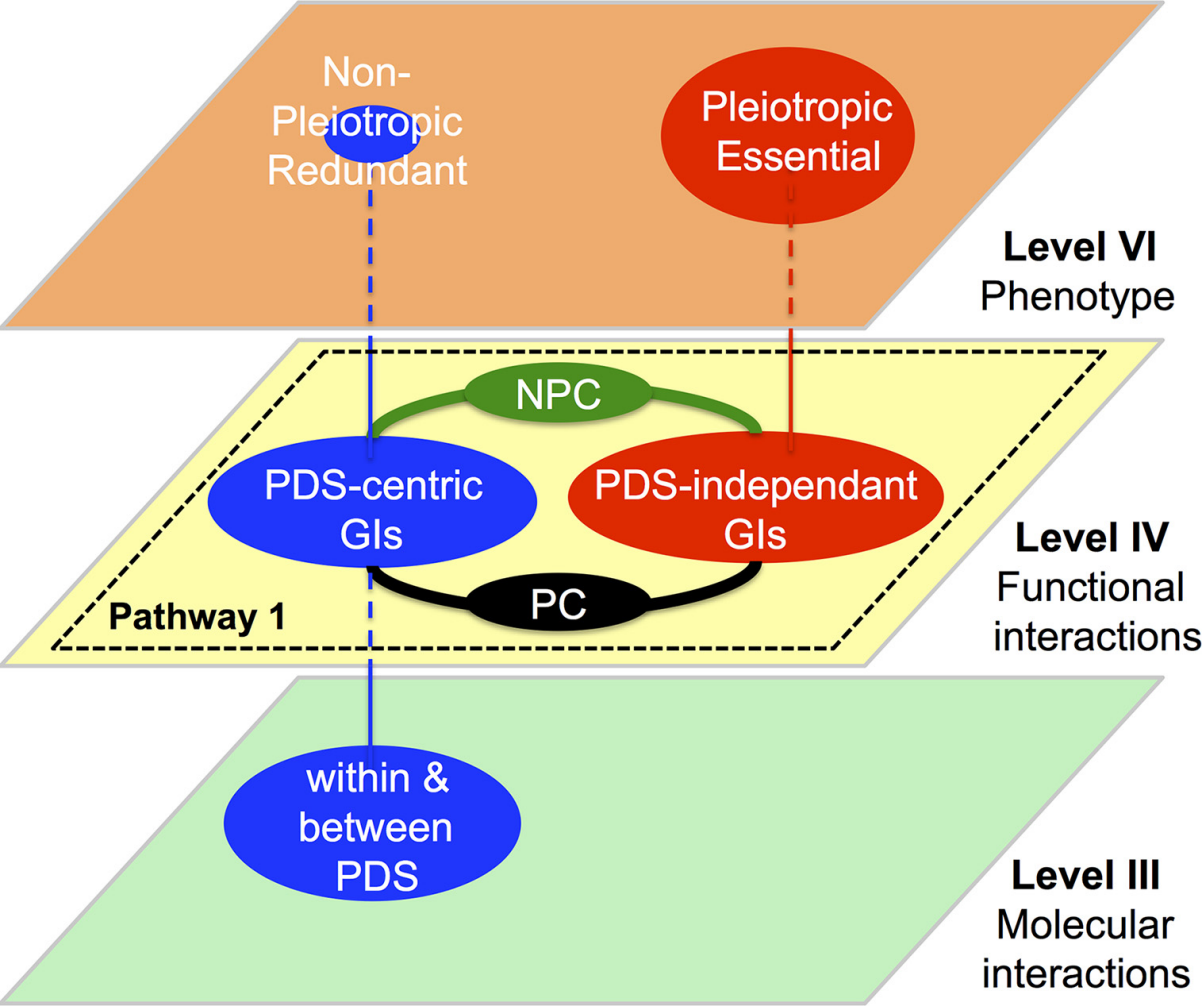
Structure and function of a GI-network within-pathway in *Caenorhabditis elegans*. Three abstraction levels are represented as plan from the bottom to top: Level III, level IV and level VI. PDS-centric interactions (C4 and C5 classes) are organized around Protein-protein interaction dense subnetworks (PDS) located at level III. They tend to involve non-pleiotropic and redundant genes (level VI). PDS-independent interactions (C1 and C2 classes) tend to involve pleiotropic and essential genes. GIs involving pleiotropic (PC; C6 class) and non-pleiotropic connectors/GI-Hubs (NPC; C3 class). They tend to link genes from an average to a high range of pleiotropy and to an average to a low rang of pleiotropy respectively.

In this study, we characterized the structure of a GI network composed of ~1,500 GIs in the nematode *Caenorhabditis elegans*. We showed that GIs form a heterogeneous group of entities with respect to attributes computed from expression, protein-protein interaction (PPI) and phenotype data. From this set of GIs, we identified six classes covering ~600 GIs, named C1 to C6 that cluster apart from gene-pairs randomly picked from the genome. Characterization of these GI classes revealed that they are either centered on protein-protein interaction dense subnetworks (PDS) and called PDS-centric interactions, or independent of these PDS (PDS-independent interactions) (Fig 9). The current study shows that PDS-centric interactions are composed of C4 and C5 GIs, and tend to involve redundant genes, particularly kinases, PPI-Hubs and non-Hub bottlenecks, which also display average to low pleiotropy (Fig 9 and S20 Fig). PDS-independent interactions are mainly composed of C1 GIs. They tend to involve essential and pleiotropic genes and also genes involved in cell division (Fig 9 and S20 Fig). Our data also suggest that PDS-centric and PDS-independent interactions coordinate gene functions within-pathways and that two classes of GIs, C3 and C6 are highly involved in this coordination due to their association with connectors/GI-Hubs. These later GI classes were shown to connect genes across ranges of pleiotropy. C6 GIs tend to connect genes with an average to genes with a high pleiotropy while C3 GIs connect genes with an average to genes with a low pleiotropy. We call GI-Hubs involved in C6 and C3 GIs, pleiotropic connectors (PC) and non-pleiotropic connectors respectively (NPC; Fig 9 and S20 Fig). Our study built a structural and functional model for GI-networks in *C*. *elegans* mainly composed of GIs within signaling and metabolic pathways. This network may be representative for a fraction of the GI network genome-wide (the union of C1-C6 GI corresponds to ~40% of GIs-all). Our data showed that GIs within C7-C10 are composed of both within and between pathway interactions that could not be distinguished from gene-pairs randomly selected from the genome using our classification strategy. Another classification strategy may consequently, be required to characterize these prominent classes of GIs. Nevertheless, our characterization of within-pathway GI classes raises critical questions relative to the coordination of genes and their protein products within pathways as well as on the role played by the individual GI classes on genomic robustness and on network evolution as discussed below.

### Coordination of molecular machines by GIs within-pathways

We showed in this study that PDS and pathways are different structural/functional modules, consistent with their respective positions at abstraction levels III (physical interactions) and IV (functional interactions) [1]. Furthermore, we showed that both PDS-centric and PDS-independent GIs contribute to pathways. While, the function of PDS and protein complexes within pathways has been extensively studied (reviewed in [42]), the function of PDS-independent GIs is quite unexplored. The apparent independence of C1 interactions towards PDS is intriguing. It may be explained by the possibility that physical interactions between protein products of genes involved in C1 GIs have not been identified yet. Interestingly, physical interactions between proteins controlling early embryogenesis, including that controlling cell division have been the subject to special attention [43], suggesting that this portion of the PPI network may be less prone to missing data than other parts. This supports the idea that cell division-associated interactions found in C1 define true PDS-independent GIs. PDS-independence of C1 GIs may also result from a potential enrichment of transcriptional regulatory elements in this class (and consequently, protein-DNA interactions instead of PPI). As an example of this possibility, C1 GIs include interactions between the chromatin modifying enzymes coding genes *mes-4*, *mes-2/3/6* and *mep-1*, which were shown to differentially modify histones and consequently, to bind to DNA and not physically to each other [44] (S6 Fig).

Our study also revealed that C3 and C6 GI classes are enriched in GI-Hubs and may play a critical role in coordinating molecular machines within pathways. GI-Hubs have been previously proposed to constitute connectors with high modifier potential–i.e. the ability to modify the expression of phenotypes resulting from genetic alterations of multiple genes–both in *C*. *elegans* and in yeast [39]. Our study also characterized C6 GIs as involving pleiotropic connectors (PC, Fig 9) and C3 GIs as involving non-pleiotropic connectors (NPC, Fig 9). The involvement of PC in the coordination of molecular machines within pathways is consistent with a study of pleiotropic genes *in C*. *elega*ns suggesting that genes involved in early embryogenesis are organized into partially overlapping functional modules and that pleiotropic genes represent connectors between these modules [45]. Our data, while supporting this model, suggest that non-pleiotropic genes may also have an organizational role within pathways through C3 interactions.

Intriguingly, genes interacting using C3 and C6 GIs, while being associated individually to several phenotypes did not present any significant correlation in their phenotypic profiles. This is the major distinctive property of C3 and C6 GIs, when compared to other classes. A study investigating the tissue specificity of PPI-network identified both housekeeping/pleiotropic and "local" PPI-Hubs [46]. It also documented the way housekeeping/pleiotropic Hubs interact with tissue-specific proteins, being consequently involved in different biological processes than other housekeeping Hubs [46]. Such a scenario transposed to GI-networks may partially explain how a connector may be associated to a panel of phenotypes which is significantly different than that of its partners.

Overall, our study establishes an organizational model for pathways suggesting that they are built around both pleiotropic PDS-independent and non-pleiotropic PDS-centric GIs coordinating molecular machines together with both pleiotropic and non-pleiotropic connectors. Such an organization suggests that mutations in genes of a given pathway may lead to either pleiotropic or non-pleiotropic effect. These effects may then be modulated at the organism or at the tissue level, through selection of compensative mutations [47] or through buffered pleiotropy effect [48]. Further characterization of the GI-network structure within and between pathways will thus certainly provide a better understanding of the abundant pleiotropy effect found in human complex diseases and traits [49].

### GI-network modularity and genomic robustness

C5 and C4 GIs involve genes with an average to a low pleiotropy. This is consistent with their enrichment in genes coding for kinases (Fig 2A) and also evolutionarily conserved redundant genes that are mostly kinases in C5 and either kinases or non-kinases in C4. While these genes may be involved in more than one bioprocess, their redundancy may highly limit the number of phenotypes expressed upon perturbation, thus reducing their apparent pleiotropy. While compensatory functions between duplicated genes were shown to be evolutionarily unstable for most of the duplicated gene-pairs [50], a small fraction of them are submitted to natural selection that stabilizes their functional overlap [45]. These selected pairs tend to have a high propensity of clustering into the same protein complexes, and share common interaction partners [45]. Protein complexes were identified form PPI networks as dense subnetworks with methods similar to that used in this study to identify PDS [51]. Altogether, these data are in agreement with C4 and C5 GIs displaying positive values for the CI attribute (gene pairs coding for proteins with a significant high number of common PPI partners; Fig 1B).They are also consistent with C4 being enriched in evolutionarily conserved redundant genes. Gene duplication, when associated to functional redundancy, was associated to genomic robustness–i.e. increased resistance of the system to genetic alterations [52]. The PDS-centric within-pathway GIs involving redundant and conserved genes within- or between-PDS may then contribute to the robustness of *C*. *elegans* genome.

### GI-network modularity and evolution

The present study shows that C1 to C6 GIs classes are enriched in within-pathway interactions while C7 to C9 classes are enriched in both within- and between-pathways GIs. Interestingly, these later GI classes could not efficiently be dissociated from gene pairs randomly picked from the genome based on the six attributes used for the classification. While attribute values may be highly influenced by missing data for these GI classes, it is intriguing to observe that the vast majority of between-pathways interactions lay in these classes. It was shown in yeast that both positive and negative GIs within functional modules (protein complexes, gene belonging to the same biological process) are significantly more conserved between *S*. *cerevisiae* and *S*. *pombe*, than wiring between these modules [19,20,53]. Considering that a pathway is a functional module, it would then be interesting to assess whether C1 to C6 GIs would be more evolutionarily conserved than C7 to C9. Similarly, it would be interesting to assess whether interactions within-PDS would be more evolutionarily conserved than between or outside PDS interactions.

The organization of the within-pathway GI-network described here is consistent with the evolution theory model called selection, pleiotropy and compensation (SPC) recently built from quantitative genetics studies (reviewed in [47]). This model predicts that adaptive change in one character (through functional alterations of a pleiotropic gene) is associated with deleterious pleiotropy in others and subsequent selections to compensate for these pleiotropic effects. This compensation involves the genetic alteration of non-pleiotropic/“private” gene(s). This model relies upon the existence of functional interaction between pleiotropic and non-pleiotropic genes within pathways targeted by evolution. Interestingly, such interactions have been identified in tissue-specific PPI-networks [46] and are also consistent with the model proposed here, in which pleiotropic Hubs are connected to genes displaying average pleiotropy through C6 GIs within pathways.

We showed that integration of attributes based on data located at different abstraction levels (in this study, levels II, III and VI), identified GI classes with distinctive biological properties from a GI-network. Similar classification strategy applied to gene-pairs found within-pathways of systems inappropriate to a genome-wide experimental mapping of GIs would be interesting to interrogate the evolutionary conservation of functional interactions in multicellular organisms. Integrative genomics approaches using a selected subset of statistical attributes may also be used to improve predictors for specific classes of GIs such as connectors/GI-Hubs, which are of high interest for health-oriented research.

### Conclusions

Our study provides the first deep structural and functional characterization of a GI-network enriched in within-pathways interactions in a multicellular organism. It proposes a model in which PDS-centric and PDS-independent interactions coordinate molecular machines within pathways together with pleiotropic and non-pleiotropic connectors. This study demonstrates the value of integrative genomics approaches, using data from several abstraction levels to characterize genetic interaction networks, their relationship with networks located at different abstraction levels and to study the systems basis of complex biological phenomena, including genomic robustness, pleiotropic effects and adaptive evolution.

## Methods

### Dataset of genetic interactions

The complete set of genetic interactions (GIs-all) consists of 1,514 GIs retrieved from Wormbase, Biogrid and/or curated manually from the literature ([54] and this study S1 Table). Five GIs were curated using the curation and blind re-curation procedure of BIOGRID [55]; 689 GIs (45%) were curated by an author-based curation approach used by Wormbase to insure the accuracy of their data [56]; 56 and 261 GIs were manually curated in our laboratory in the context of [54] and this study respectively (S1 Table). The 261 GIs curated in this study cover GIs identified from the literature by Textpresso [22] and found in GI classes (C1 to C6) (S1 Table). References to an experimental validation were found in the literature for 252 of these GIs, leaving nine of them without experimental evidences (3.57%). Less than 3.3% of GIs lay in that category of non-validated interactions per class. GIs-all was used to build a GI network analyzed and visualized using Cytoscape v2.8.2 [57]. GI degree was computed for each gene using the network statistic tool.

### Attributes used to cluster GIs into classes

Attributes and data used to compute them have been described previously [54]. Briefly, The co-expression attribute *Exp*(A, B) is the *P*-value derived for the Pearson correlation of genes A and B across 514 microarray experiments (retrieved from [58]) relative to the empirically estimated probability distribution of correlation for all gene pairs (i.e. a fitted normal). The co-phenotype attribute *Ph*(A, B) uses 107 phenotypes (retrieved from WormBase release WS141) and measures the statistical significance of the number of shared phenotypes between the two genes (A and B) via a standard Fisher’s exact test (N = the number of phenotypes observed for at least two genes). The multispecies PPI network was taken from [54]. Briefly, PPIs were obtained from all *C*. *elegans*, *Saccharomyces cervisiae*, *Drosophila melanogaster*, and *Homo sapiens* yeast two-hybrid datasets stored in BioGRID v2.0.37 (http://www.thebiogrid.org/) and from two additional yeast two-hybrid datasets [59,60]. To create a multi-species PPI network, we used the orthology mappings generated by InParanoid v1.35 [61] (non-default parameters: score cutoff 10, in-paralog confidence cutoff 0.025, sequence overlap cutoff 0.2). The interaction attribute *I*(A, B) indicates whether the proteins encoded by A and B exhibit a PPI in the multi-species PPI network as defined in [54]. Similarly, the common interactors attribute, *CI*(A, B), considers the statistical significance of the observed number of common physical interactors of the proteins encoded by A and B, in the multi-species PPI network. The attribute *CI* is then assigned a *P*-value derived from a one-tailed Fisher’s exact test (N = the number of genes encoding proteins that are in the multi-species PPI network). For the other attributes, a biological network called the PhEP was created where two genes A and B are connected by an edge if the Pearson correlation coefficient of their gene expression exceeds 0.35 or if their gene products exhibit a PPI in the multi-species PPI network. For both A and B, we measured how surprising it is to witness the observed number of their neighbors (i.e. genes connected to it by one edge) in the PhEP network labeled with a specific phenotype identified by RNAi in *C*. *elegans*. This was measured using a one-tailed Fisher’s exact test (N = the number of genes with some assigned phenotype). If the derived *P-*value is less than or equal to 0.05 for A and B for at least one phenotype, we assign a value of 1 to a categorical variable *N*(A, B), and 0 otherwise. Similarly, if A and B exhibit a phenotype that is also enriched in both their neighborhoods in the PhEP network, a value of 1 is assigned to a categorical variable *NPh*(A, B), and 0 otherwise. Missing values for any of the derived attributes (due to missing values in the underlying datasets) were replaced with the expected value (i.e. the sample mean) of the attribute before training. See [54] for additional information regarding the attributes and datasets used to derive them.

### Heatmaps

Unless otherwise specified, heatmaps were the result of hierarchical clustering using Ward’s agglomerative method with a distance metric between *x* and *y* based on Euclidean distance and is given by:

$$d=\sqrt{{\sum_{i}\left(x_{i}-y_{i}\right)}^{2}}$$

The Canberra distance is given by:

$$d_{x,y}=\sum_{i}\frac{\left|x_{i}-y_{i}\right|}{\left|x_{i}\right|-\left|y_{i}\right|}$$

where *d*_*x*,*y*_ is the Canberra distance between two GI classes *x* and *y*, *x*_*i*_ and *y*_*i*_ is the number of genes that occur in the classes. The binary distance is defined as the fraction of the *n* genes that is present in only one of classes *x* and *y*.

### GI classes identification

GIs-all was hierarchically clustered, and uncertainty in the resulting clusters was judged with approximately unbiased (AU) *P-*values acquired after a multiscale bootstrap resampling of the data (10,000 resamples) using the R package “pvclust” v1.2–2 with the default parameters [62]. The dendrogram was cut at each height with the R function cutree. Clusters with AU >0.95 were retained for further analysis. For each remaining cluster of genetic GIs (positive set), a logistic regression model was tested using leave-one-out cross-validation (LOOCV) against a negative set (randomly selected gene pairs) of equal size with the requirement that one of the genes found in a negative pair was included in the cluster of genetic interactions. For all clusters, the six attributes were used in the regression models. True and false positive rates were computed at 20 equally spaced model score cutoffs in [0,1], resulting in a receiver operating characteristic (ROC) curve for each model. The area under the ROC curve (AUC) was used as an indicator of how well a classifier model could discriminate GIs found in our positive training sets from negative examples.

### Enrichment calculation

Gene Ontology (GO) term, essential gene, redundant genes, PPI-Hubs, GI-Hubs and High-PI genes enrichments were evaluated using a one-tailed Fisher’s exact test. Measurement of enrichment in groups of GIs defined by positive or negative values for a single attribute required the identification of a threshold above which the attribute value is considered as positive. These thresholds are indicated in the S6 Table as well as the number of GIs within GIs-all associated to a positive value to the attribute. For GO term enrichments, the reference universe *N* was constituted of all terms associated to genes (with or without repetition) found in genetic interactions of GIs-all (see Dataset of genetic interactions section). Note that certain genes are involved in more than one GI within GI classes. As indicated in the result section, GO enrichment was done considering the gene frequency within classes (each repetition being considered as an independent gene) or without considering the gene frequency (each gene is used only once per class and in GIs-all to calculate the enrichment). The universe *N* for the other enrichment tests contained all genes (with repetition) found in GIs-all.

### Monochromaticity index

To evaluate the monochromatic index of each GI class, we first partitioned the GIs-all network into several dense subnetworks using the Cytoscape plugin “MINE” v1.5 [37] with the default parameter values. The resulting network contained a total of 42 GIs subnetworks (GDS) (S3A Fig). To assess the proportion of interactions from a GI class within a particular GDS, we calculated a monochromatic score (MS) in a similar way than described previously [6]. Let *B*_*R*_ represent the ratio of GIs from a given class within GIs-all and *M*_*R*_, the ratio of GIs from the same GI class within a GDS. The monochromatic scores of a GI class and for a GDS is given by:

$$\begin{array}{l} if\;M_{R}>B_{R},MS=\frac{\left(M_{R}-B_{R}\right)}{\left(1-B_{R}\right)}\\ if\;M_{R}=B_{R},MS=0\\ if\;M_{R}<B_{R},MS=\frac{\left(M_{R}-B_{R}\right)}{B_{R}} \end{array}$$

### Protein-protein interaction (PPI) network analysis

PPI-Hubs were identified as the 20% proteins with the highest PPI-degree (k ≥ 22) as previously done [36]. The degree and the betweenness centrality were assessed for all genes, Hubs and non-Hubs using the network statistic tool in Cytoscape v2.8.2 [57]. Distribution of interaction degrees and betweenness centralities was computed for all the genes (considering the frequency of involvement for each gene in GIs of the class) in a given set of GIs.

### PPI dense subnetworks and pathways

The modular partitioning of PPI-networks was done using the Cytoscape plugin “MINE” v1.5 [37] with the default parameter values. Significant PPI dense subnetworks (PDS) were selected by taking all complexes with a score (Density * Number of Proteins) ≥ 4, giving a total of 106 PDS covering 1,760 proteins connected by 17,430 edges. The size distribution of all PDS is given in S14A Fig. Significant GI-dense modules (GI-modules) were selected by taking all complexes with a score (Density * Number of Proteins) ≥ 3. KEGG pathways were retrieved from the Kyoto Encyclopedia of Genes and Genomes database 61.1 release [41]. Pathways from the literature were manually curated from [63] (S3 Table).

### Within- and between- PDS/pathway assessments

To measure the enrichment of within-PDS/pathway and between-PDS/pathway within classes of GIs and pathways, we defined several networks. Networks *U*_*Ci*_ are composed of genes and interactions found in GI classes C*i*. A network *W*_*Pa*_ is composed of genes found in pathways, which are linked by an edge if these genes are found in at least one common pathway. We also defined a network W_PDS_, which is composed of proteins and PPI found within PDS. The networks B_Pa_ and B_PDS_ are composed of nodes found in W_Pa_ and W_PDS_ respectively and all possible edges between these nodes from which were removed the edges found respectively in W_Pa_ and W_PDS._ (note that B_PDS_ do not overlap with the PPI-network). We then computed the frequency *F* in these networks with respect to the mean frequency calculated for a random network *V* as follows:

$$F_{X,V}=\frac{\sum \left(T_{X}\cap T_{Y}\right)}{\sum T_{X}}/\frac{\sum \left(T_{V}\cap T_{Y}\right)}{\sum T_{V}}$$

where *T*_*X*_, *T*_*Y*_ and *T*_*V*_ represent all edges in network *X*, *Y* and *V* respectively.

To compute the frequency of within-PDS and between-PDS interactions found in pathways, we defined the following: X = W_Pa_, Y = W_PDS_ and B_PDS_ respectively, and V is a random network with the same structure as W_Pa_ (detailed in the Network randomization section below).

To compute the frequency of within-PDS and between-PDS found in GI classes, we defined the following: X = U_Ci_. Y = W_PDS_ and B_PDS_ respectively, and V is a random network with the same structure as U_Ci_.

To compute the frequency of within-pathways and between-pathways found in GI classes, we defined the following: X = U_Ci_, Y = W_Pa_ and B_Pa_ respectively and V is a random network with the same structure as U_Ci_.

We then computed the log_10_-ratio (*LR*) transform score of the frequency for network *X* relative to randomized network *V*. To avoid the log-ratio of a zero value, we used a simple transform that took care of any undetermined possibilities as follows:

$$LR\left(F_{X,V}\right)=|\begin{array}{l} {\text{log}}_{10}\;if\;F_{X},F_{V}>0\\ -1\;if\;F_{X}>0\&F_{V}=0\\ 0\;if\;F_{X}=0\&F_{V}=0 \end{array}$$

### Network randomization

The following randomization procedure was used to randomize GI networks (Figs 4 and 6 and S13 Fig) and pathways (Fig 5). To do so, for each network being randomized, all connected gene-pairs were split in two groups. The order of genes and the number of edges in the first group were kept unchanged. Genes in the second group were randomly reordered. This aims to preserve the degree of connectivity for any gene present in the network and its randomized version. Restriction was applied to make sure that a pair of gene could not be composed of twice the same gene. The number of randomized networks generated was determined giving Hoeffding’s inequality [64]. Basically, by increasing the number (*n*) of random networks, we minimize the relative error *ρ* and get a better estimation of *p*, the real mean frequency of edges in within- or between-pathways/PDS. Since the calculated mean *μ* is an estimation of the real mean *p* and ε=ln2−ln(1−c)2n, let *μ*^−^ = max{0,*μ*−*ε*} and *μ*^+^ = max{1,*μ*+*ε*} and with probability *c* = 0.99, *μ*^-^ < *p* < *μ*^+^. And if *μ*^-^ > 0, we get with probability *c*, the maximum value of *ρ*:

$$\rho =\frac{\left|\mu -p\right|}{p}\leq \frac{\text{max}\{\left|\mu -\mu^{-}\right|,\left|\mu -\mu^{+}\right|\}}{\mu^{-}}$$

For all networks being randomized, 100,000 randomizations were sufficient to obtain a reasonably small value of the relative error *ρ* with a probability of 0.99. As a consequence, error bars cannot be seen (because they are too small) and are not indicated on bar graphs in Figs 4–6.

The second randomization method, used to validate within- and between-PDS relationships (S14 Fig), aimed at randomizing all the PDS node labels to create new PDS (Random; S14D Fig) with the exact same topology than that extracted from the PPI network (Original; S14C Fig), but with different node labels. In short, for a given PDS, we permuted the node labels by randomly selecting labels from a list of nodes present in another PDS and not already been reassigned. The procedure was done iteratively until more than 90% of labels in a given PDS were permuted. Edges were unchanged to preserve the degree distribution, PDS size, within- and between-PDS connectivity (as seen in S14C and S14D Fig). After the randomization process, the resulting network contained less than 3% overlapping edges with the original PDS newtork. Note that all PPI used in our study have been generated using yeast two-hybrid systems which use protein bait to identify preys. However, the bait/prey orientation of PPIs was not considered in this study.

### Distribution pleiotropic indices (PIs)

IDs of observed phenotypes for every gene found in the *C*. *elegans* genome, and their hierarchical relationships, were downloaded from WormBase (release WS220-bugfix). Relationships between phenotypes were visualized in Cytoscape, where a node represents a phenotype, and an edge between two nodes, the hierarchical relationship between two phenotypes. Groups of phenotype corresponding to the 22 most general phenotypes, covering in 1 step the entire network, were identified (S15 Fig). The pleiotropic index (PI) of a gene was computed as the number of these 22 classes containing at least one phenotype associated with the gene. This strategy was used to ensure that we identify the involvement of a gene in different tissues and at different developmental stages without being biased by the extensive characterization of certain developmental stages and/or biological processes. As seen in S15 Fig, some groups of phenotypes, for example, "Developmental variant" and "morphology variant" are associated to a much larger number of specific phenotypes than other groups. Several specific phenotypes of highly populated groups may be attributed to individual genes. Our strategy avoids having those genes being pleiotropic if not associated to other phenotypic groups.

Each distribution of PIs was computed from a given set of genes, e.g. all *C*. *elegans* genes, or genes in a given set of GIs. Odds ratios (*OR*), used to measure the enrichment of GIs between genes within or across certain PI ranges, are defined as:

$${\text{log}}_{10}\left(OR\right)={\text{log}}_{10}\left[\frac{n_{x,i}/n_{all,i}}{N_{x}/N_{all}}\right]$$

where *n*_*x*,*i*_ = number of class *x* GIs (e.g., C1 GIs) in subnetwork *i* (*i* being for example a subnetwork of GIs between genes with pleiotropic index >8); *n*_*all-i*_ = total number of GIs in subnetwork *i*; *N*_*x*_ = total number of class *x* GIs (e.g. C1 GIs) and *N*_*all*_ = total number of GIs in the genetic interactome. Significant enrichments of GI classes in each subnetwork were estimated using a one-tailed Fisher’s exact test. Only *OR* associated with a *P*-value <0.05 were represented in S17 Fig.

### Wilcoxon rank-sum test

Wilcoxon rank-sum test was done according to Hollander and Wolfe (1972) using the wilcoxon.test function in R. This test is used when the population cannot be assumed to be normally distributed.

### *P*-values

All computed *P*-values were adjusted using the Benjamini and Hochberg (1995) method for controlling the false discovery rate (specifically, with the p.adjust function in R).

## Supporting Information

S1 Fig Cluster selection within GIs-all. The x-axis shows the number of clusters with approximately unbiased *P*-value (higher values indicate greater significance, see Methods) greater than or equal to the threshold indicated in the parentheses. The y-axis shows area under the curve (AUC) values following cross-validation analysis of each cluster-based model. Each dot represents one cluster and its magnitude represents the number of GIs within the cluster. Dots inside the red rectangle correspond to the 6 clusters selected for further analysis based on their high AUC values and comparable sizes.
(TIF)

S2 Fig Genetic interaction classes and associated missing data. Percentage of interacting gene pairs missing co-expression, phenotype or protein-protein interaction data are indicated.
(TIF)

S3 Fig GI classes tend to form GDS in a biased manner. **(a)** Hierarchical clustering of monochromatic indices of GI classes. Each row represents a GDS extracted from GIs-all using the MINE tool. GDS sizes are indicated by the blue shaded legend. **(b)** Enrichments of GIs classes and pair combinations of classes in GDS when compared to GIs-all. Only the statistically significant enrichments are shown with -Log_10_ adjusted *P*-values (*P* < 0.05, see Methods). The GDS size indicates the total number of GIs (edges) of the subnetwork.
(TIF)

S4 Fig Hierarchical clustering of GI classes based on their genes frequencies. Gene frequencies are clustered using Binary (left dendrogram) and Canberra (right dendrogram) distance metrics (see Methods).
(TIF)

S5 Fig Examples of genetic interactions from C1 to C6 classes. Nodes represent genes connected by genetic interactions from the six classes of GIs. Are also indicated, protein-protein interaction dense subnetworks (PDS) and genes involved in C1 to C6 GIs and also involved in signalling pathways controlling vulval development (S3 Table).
(TIF)

S6 Fig Genetic interactions found in C1 GIs. Refer to S5 Fig for the nodes and edges descriptions.
(TIF)

S7 Fig Genetic interactions found in C2 GIs. Refer to S5 Fig for the nodes and edges descriptions.
(TIF)

S8 Fig Genetic interactions found in C3 GIs. Refer to S5 Fig for the nodes and edges descriptions.
(TIF)

S9 Fig Genetic interactions found in C4 GIs. Refer to S5 Fig for the nodes and edges descriptions.
(TIF)

S10 Fig Genetic interactions found in C5 GIs. Refer to S5 Fig for the nodes and edges descriptions.
(TIF)

S11 Fig Genetic interactions found in C6 GIs. Refer to S5 Fig for the nodes and edges descriptions.
(TIF)

S12 Fig Protein-protein interactions degree distribution for the multi-species *Caenorhabditis elegans* interactome. PPI-Hubs are identified as the top 20% most connected proteins in the PPI network indicated by a dashed line.
(TIF)

S13 Fig C7 to C9 classes are enriched in between-pathways interactions. Log-Ratio scores for within-pathway (red bars) and between-pathway (blue bars) relationships observed between genes interacting through unselected GI classes (C7-C10) or present in GIs-all. A positive Log-Ratio score means that the frequency of within- or between-pathway GIs occurring in GI classes is significantly higher than the frequency of similar situations witnessed in relevant randomized GI networks with a probability of 0.99.
(TIF)

S14 Fig Enrichment of within- and between-PDS relationship within pathways is not significantly influenced by the topology of PDS. (a) Size distribution of 106 protein-protein interaction dense subnetworks (PDS). (b) Log-Ratio scores for within-PDS and between-PDS relationships occurring within-pathways. Different PDS networks were built by varying the number (*N*) and size (*S*) of the PDS. The original network "All" correspond to the union of PDS used for the study presented in Fig 6. Mean of Log-Ratio are indicated for Randomized PDS networks (Rdm) (n = 100) have the exact same topology than the original PDS network. Error bar indicate standard deviation of Log-ratio obtained across the 100 Rdm. (c-d) Depiction of the original and the random PDS networks topology.
(TIF)

S15 Fig Representation of the 22 classes of phenotypes identified in *C*. *elegans*. Phenotype IDs retrieved from WormBase (release WS220-bugfix) are represented by nodes and their hierarchical relationships represented by edges. Groups of phenotypes corresponding to the 22 most general phenotypes, and their first neighbors in the network were identified by different node colors.
(TIF)

S16 Fig Distribution of GI degree and pleiotropic index (PI). **(a)** Degree distribution for GI degrees in GIs-all. GI-Hubs, corresponding to the 20% GIs with the highest GI degrees, are located on the right side of the dashed line (GI degree ≥ 16). **(b)** PI distribution for all genes with PI > 0 in the *C*. *elegans* genome and for all interacting genes in GIs-all. High-PI genes, corresponding to the 20% genes with the highest PI, are located on the right side of the dashed line (PI ≥ 6).
(TIF)

S17 Fig Interaction between gene within or across pleiotropic ranges. Log odds ratios of GI classes enriched in interactions between genes displaying the same range of PI higher or equal to a given threshold (τ) (upper panel); or lower or equal to τ (middle panel). GI classes enriched in interactions in which one partner (gene A) displays a PI ≥ τ and the other (gene B) display a PI < τ are also indicated. τ is indicated by the x-axis. Only significant enrichments of GI classes are indicated (Fisher’s exact test, *P* < 0.05). High log odds ratio indicates high enrichment of GI classes within or between indicated PI ranges.
(TIF)

S18 Fig GI groups and GI classes enrichments for different biological characteristics. Enrichment in GI classes and GI groups of Genetic interaction-Hubs (GI-Hubs), redundant genes, protein-protein interaction Hubs (PPI-Hubs), Highly pleiotropic genes (High-PI) and essential genes. -Log of *P*-values from Fisher’s exact test are indicated. GI groups are associated to a positive value (+; above a threshold) or a negative value (-; below the threshold) for indicated attributes. Threshold values for each attributes are indicated S6 Table.
(TIF)

S19 Fig Log-Ratios for GI groups and GI classes occurring within or between PDS or pathways. Log-Ratios profiles for GI classes and GI groups associated to a positive (+) or a negative (-) values for indicated attributes. Blue boxes indicate enrichment of biological characteristics for C4 and C5 GI classes and CI(+) GI group.
(TIF)

S20 Fig Summary of the distinctive characteristics identified for modules and connectors. (TIF)

S1 Text Supplementary Information. (PDF)

S1 Table GIs-all with selected GI classes. (See Methods.)
(XLSX)

S2 Table Gene Ontology terms enriched amongst genes in GI classes. (XLSX)

S3 Table *C*. *elegans* signaling pathways curated from the literature. (See Methods.)
(XLSX)

S4 Table Genes involved in the same pathway and either involved in the same PDS or in different PDS. (XLSX)

S5 Table Log odds ratio and hypergeometric test *P*-value relative to S17 Fig. (XLSX)

S6 Table GI groups. Thresholds used to identify GI groups associated to a positive or negative value for attributes as well as the size of each group.
(TXT)
