# Supplementary figures and images for: Structural and Functional Characterization of a Caenorhabditis elegans Genetic Interaction Network within Pathways
Source: PLoS Comput Biol. 2016 Feb 12;12(2):e1004738. doi: 10.1371/journal.pcbi.1004738 (PMC4752231; doi:10.1371/journal.pcbi.1004738)

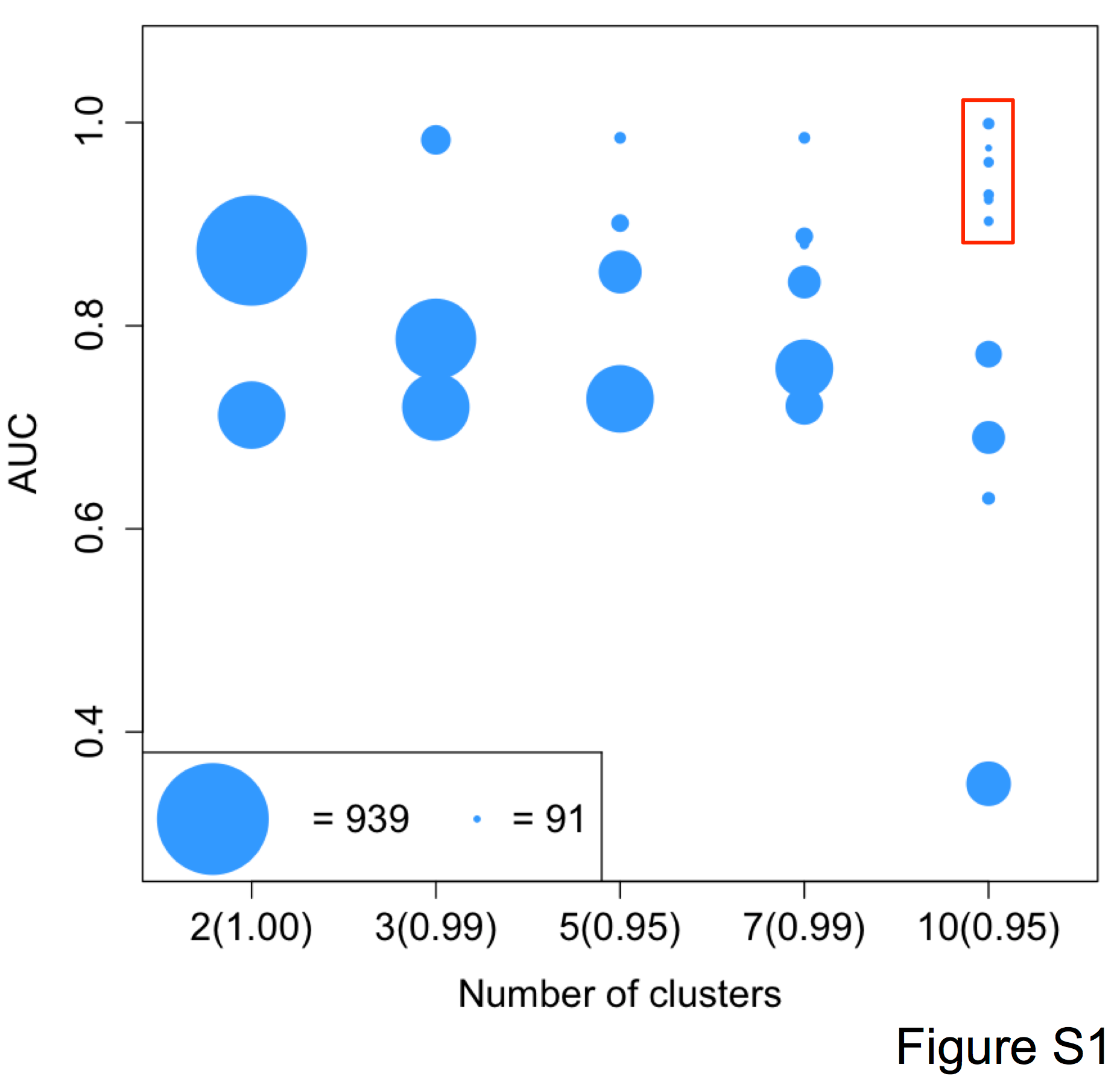

Supplement: S1 Fig — The x-axis shows the number of clusters with approximately unbiased P-value (higher values indicate greater significance, see Methods) greater than or equal to the threshold indicated in the parentheses. The y-axis shows area under the curve (AUC) values following cross-validation analysis of each cluster-based model. Each dot represents one cluster and its magnitude represents the number of GIs within the cluster. Dots inside the red rectangle correspond to the 6 clusters selected for further analysis based on their high AUC values and comparable sizes. (TIF) [file pcbi.1004738.s001.tif]

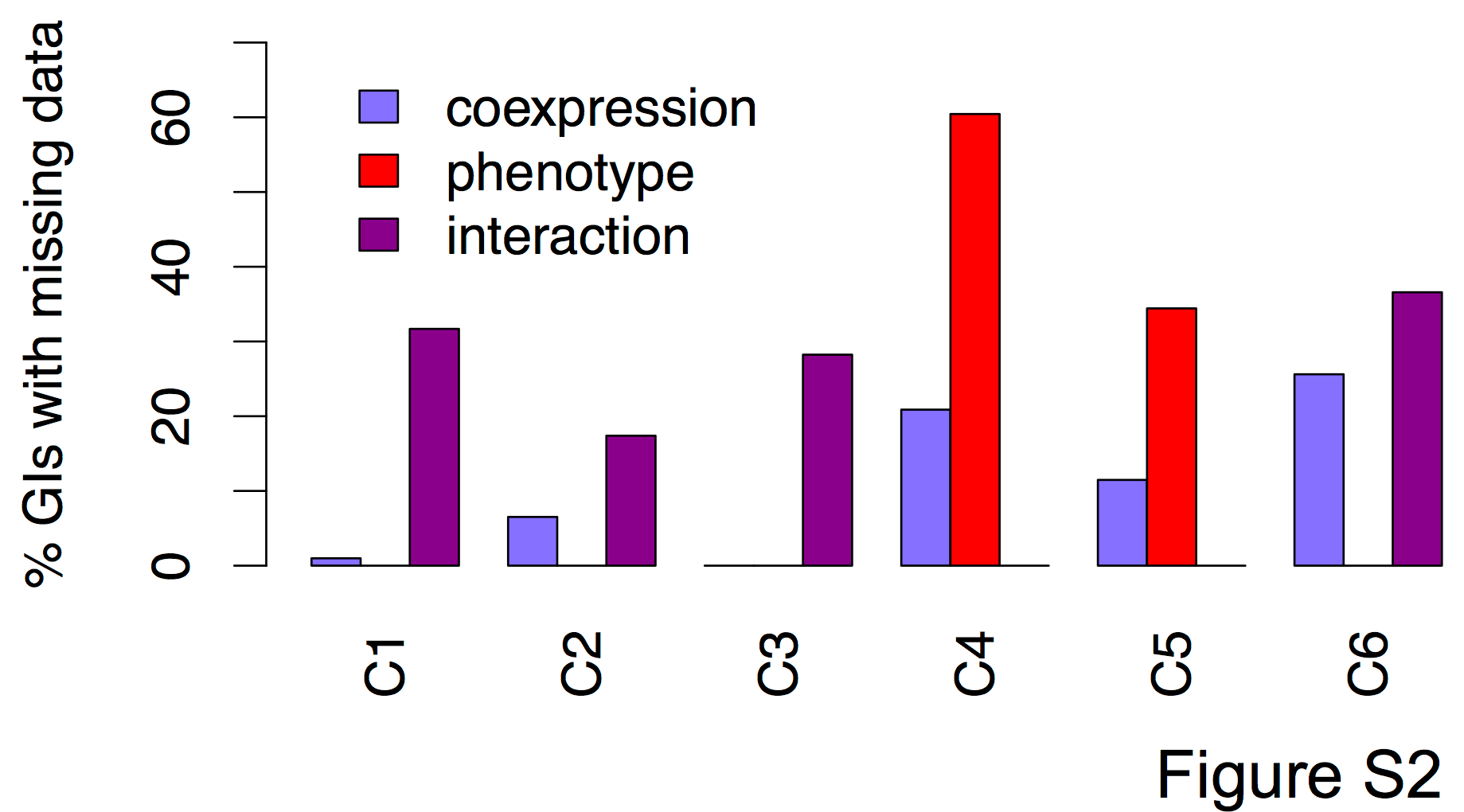

Supplement: S2 Fig — Percentage of interacting gene pairs missing co-expression, phenotype or protein-protein interaction data are indicated. (TIF) [file pcbi.1004738.s002.tif]

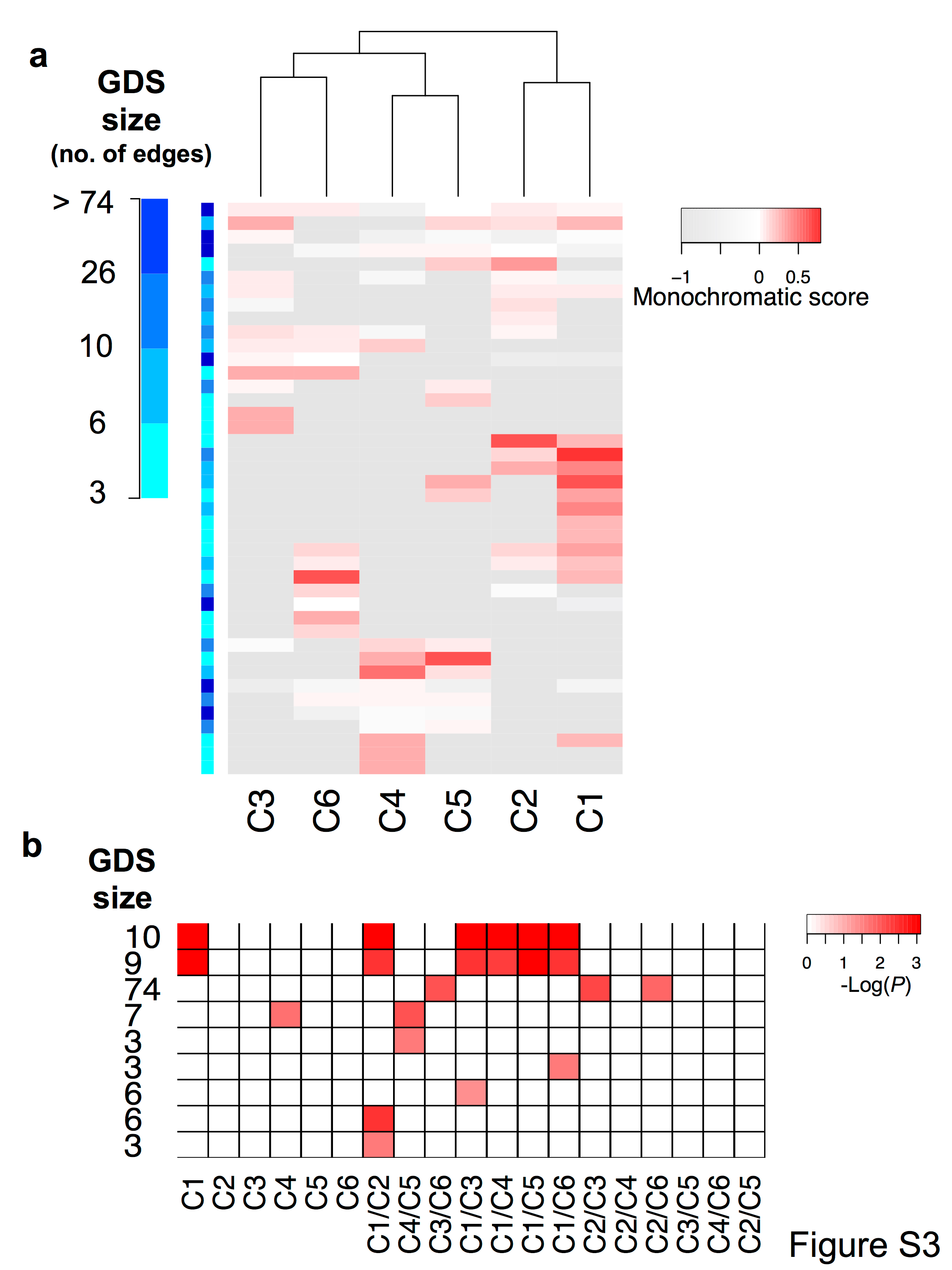

Supplement: S3 Fig — (a) Hierarchical clustering of monochromatic indices of GI classes. Each row represents a GDS extracted from GIs-all using the MINE tool. GDS sizes are indicated by the blue shaded legend. (b) Enrichments of GIs classes and pair combinations of classes in GDS when compared to GIs-all. Only the statistically significant enrichments are shown with -Log10 adjusted P-values (P < 0.05, see Methods). The GDS size indicates the total number of GIs (edges) of the subnetwork. (TIF) [file pcbi.1004738.s003.tif]

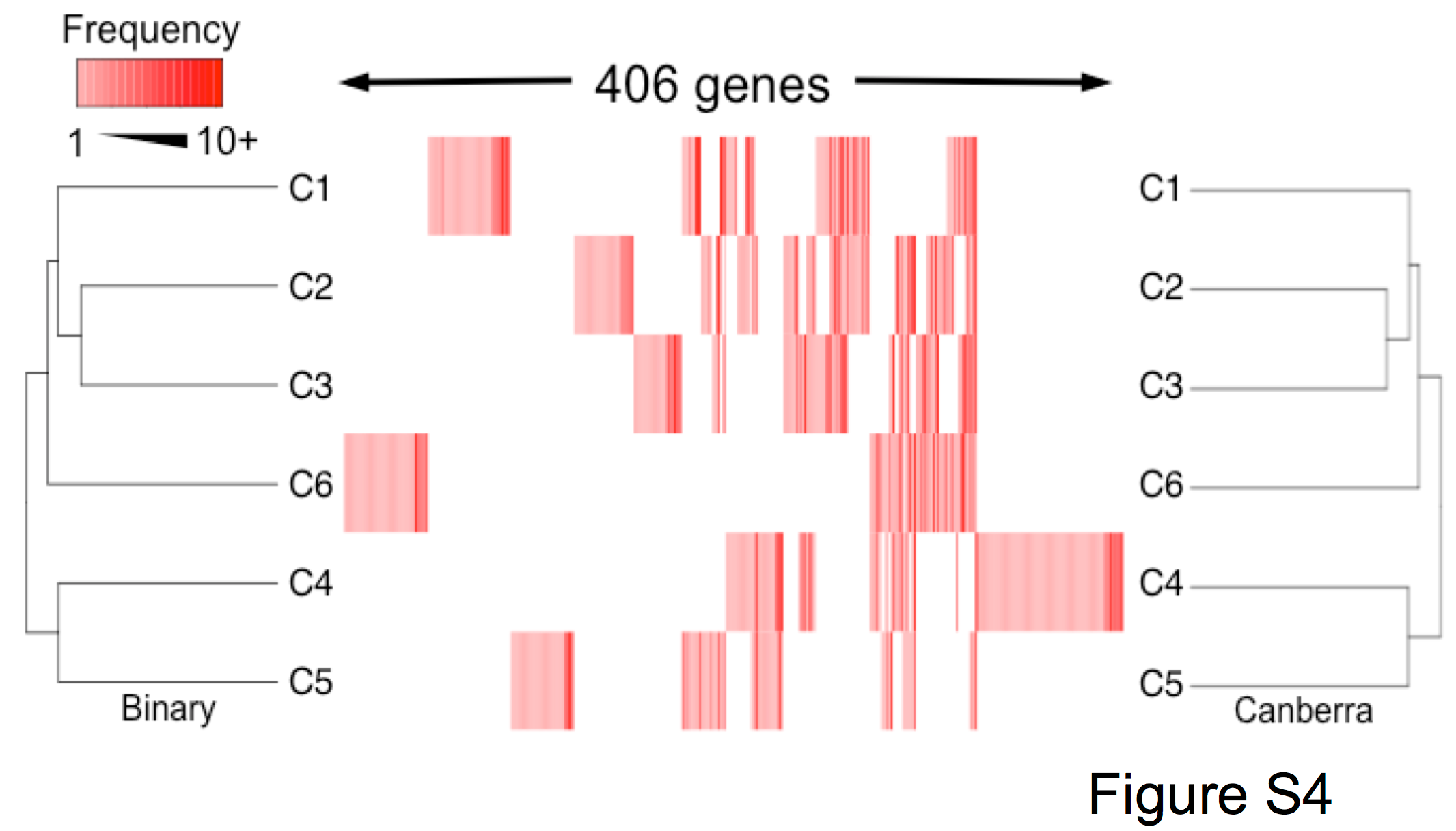

Supplement: S4 Fig — Gene frequencies are clustered using Binary (left dendrogram) and Canberra (right dendrogram) distance metrics (see Methods). (TIF) [file pcbi.1004738.s004.tif]

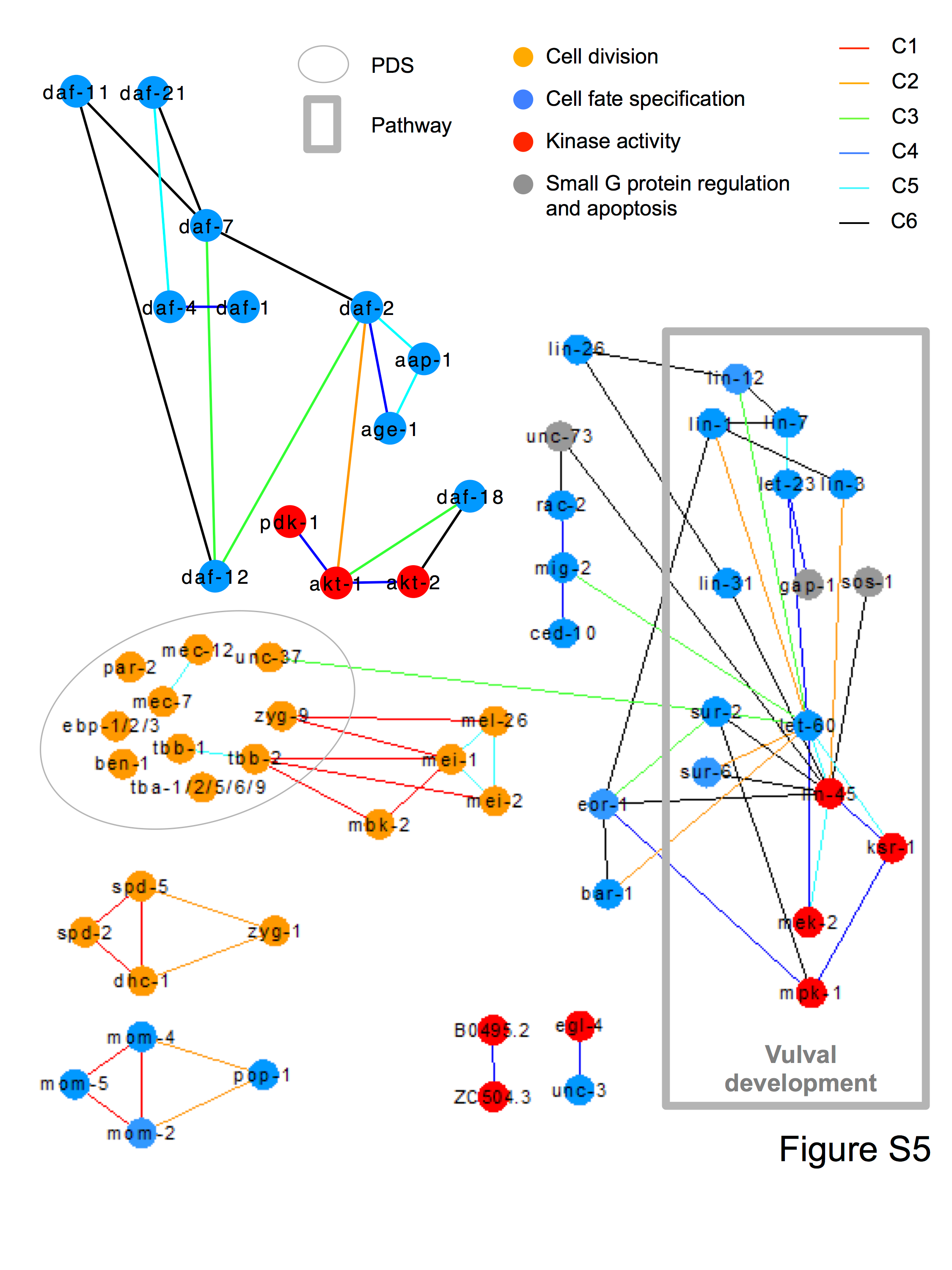

Supplement: S5 Fig — Nodes represent genes connected by genetic interactions from the six classes of GIs. Are also indicated, protein-protein interaction dense subnetworks (PDS) and genes involved in C1 to C6 GIs and also involved in signalling pathways controlling vulval development (S3 Table). (TIF) [file pcbi.1004738.s005.tif]

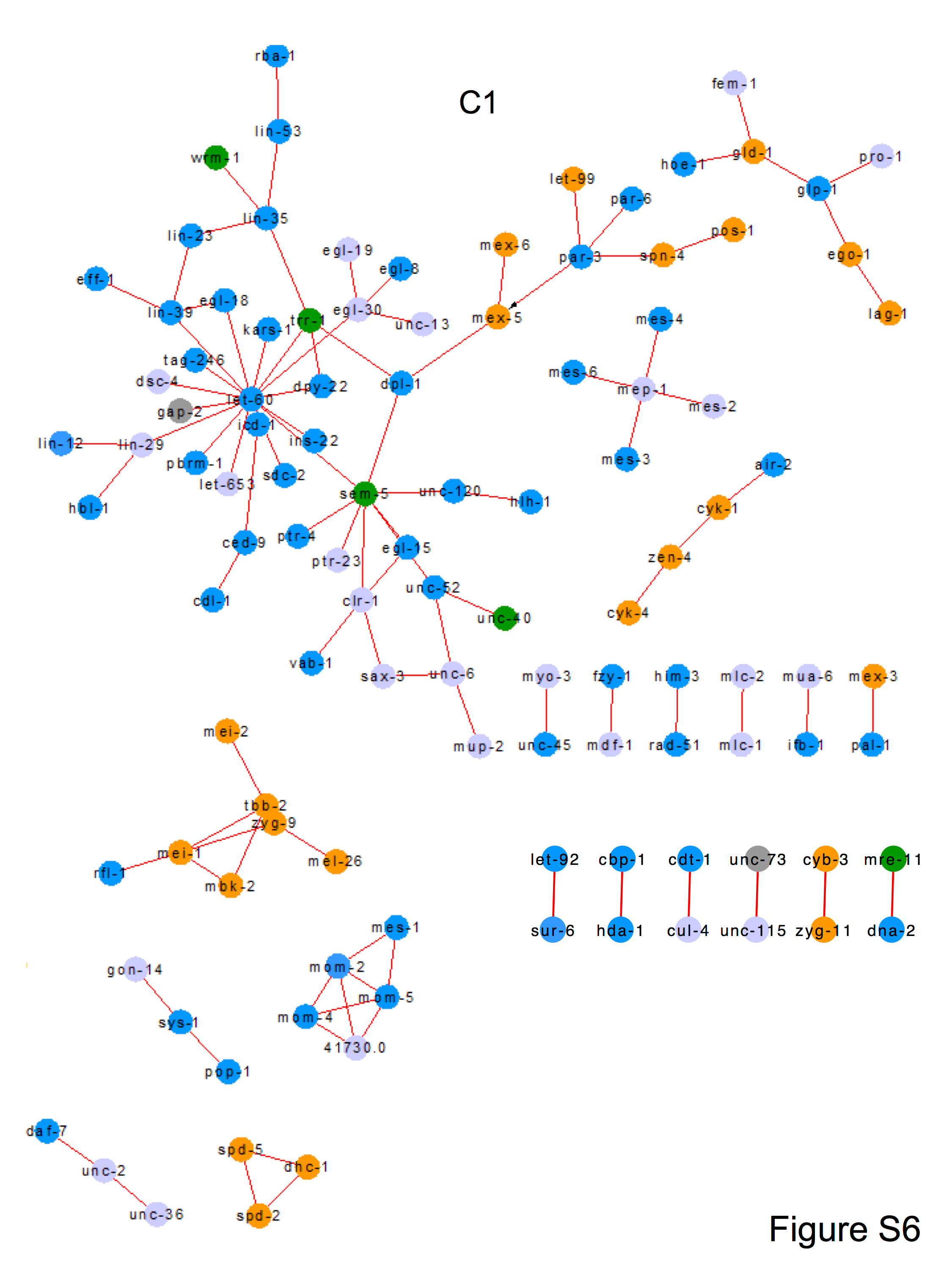

Supplement: S6 Fig — Refer to S5 Fig for the nodes and edges descriptions. (TIF) [file pcbi.1004738.s006.tif]

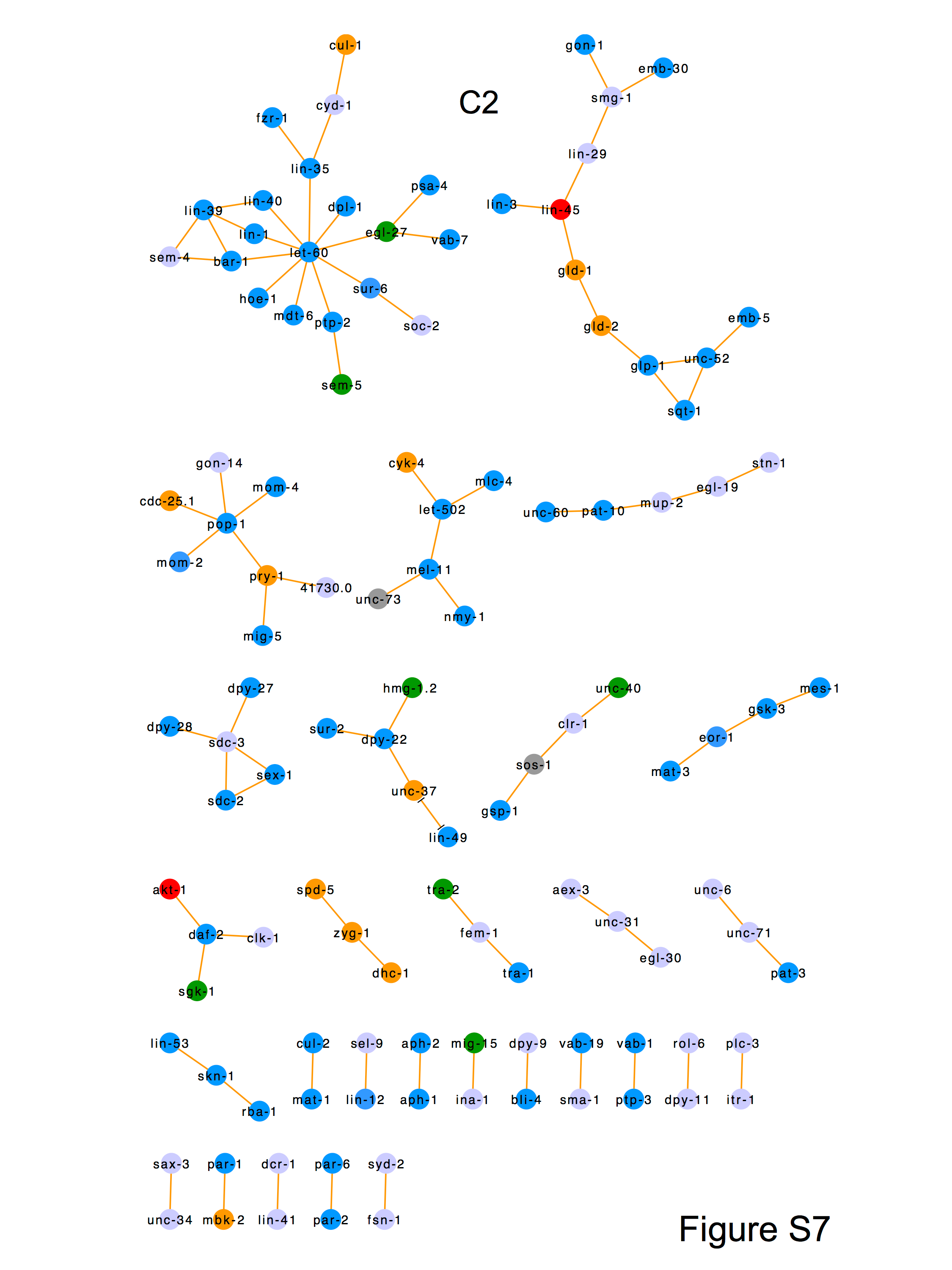

Supplement: S7 Fig — Refer to S5 Fig for the nodes and edges descriptions. (TIF) [file pcbi.1004738.s007.tif]

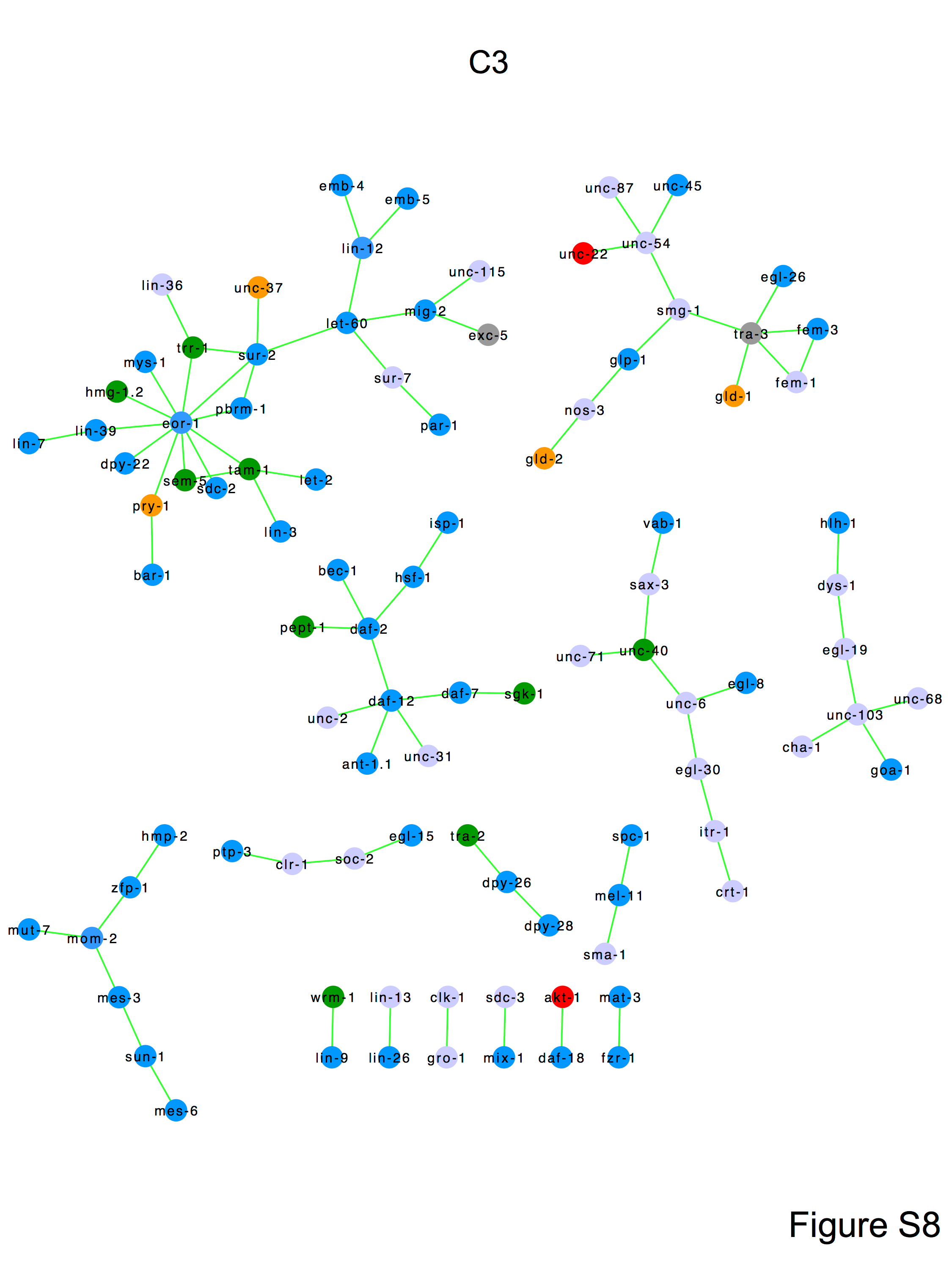

Supplement: S8 Fig — Refer to S5 Fig for the nodes and edges descriptions. (TIF) [file pcbi.1004738.s008.tif]

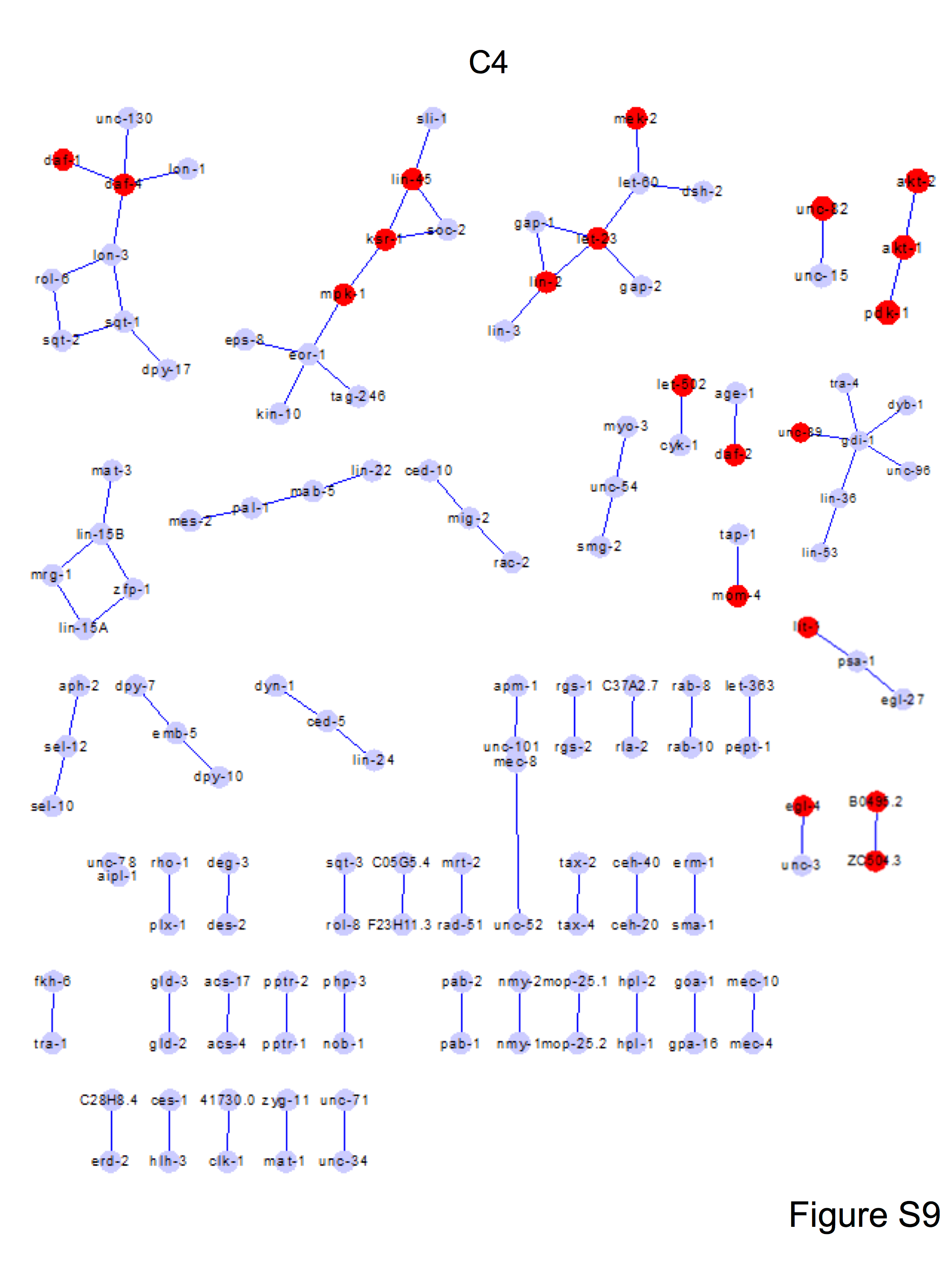

Supplement: S9 Fig — Refer to S5 Fig for the nodes and edges descriptions. (TIF) [file pcbi.1004738.s009.tif]

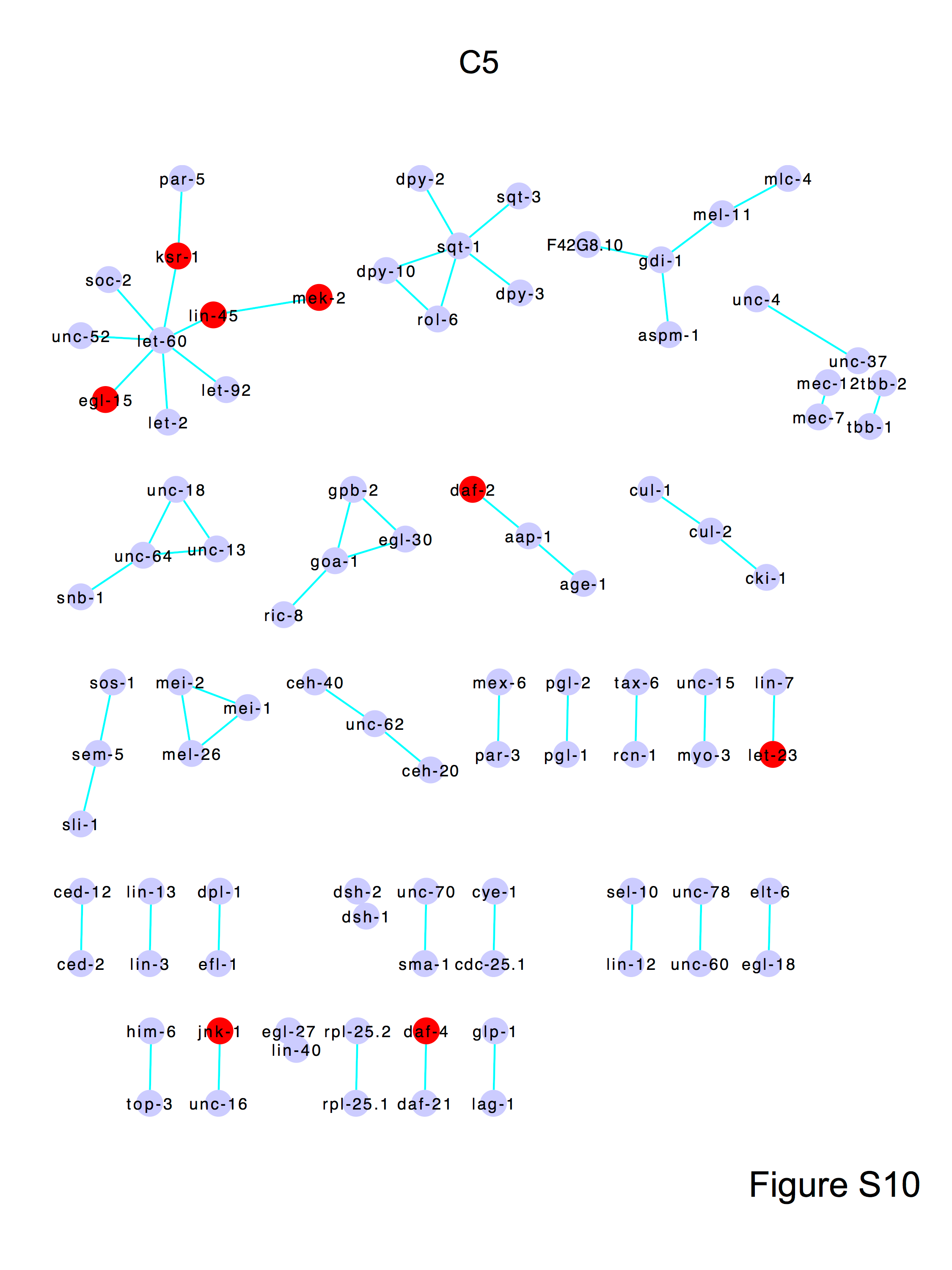

Supplement: S10 Fig — Refer to S5 Fig for the nodes and edges descriptions. (TIF) [file pcbi.1004738.s010.tif]

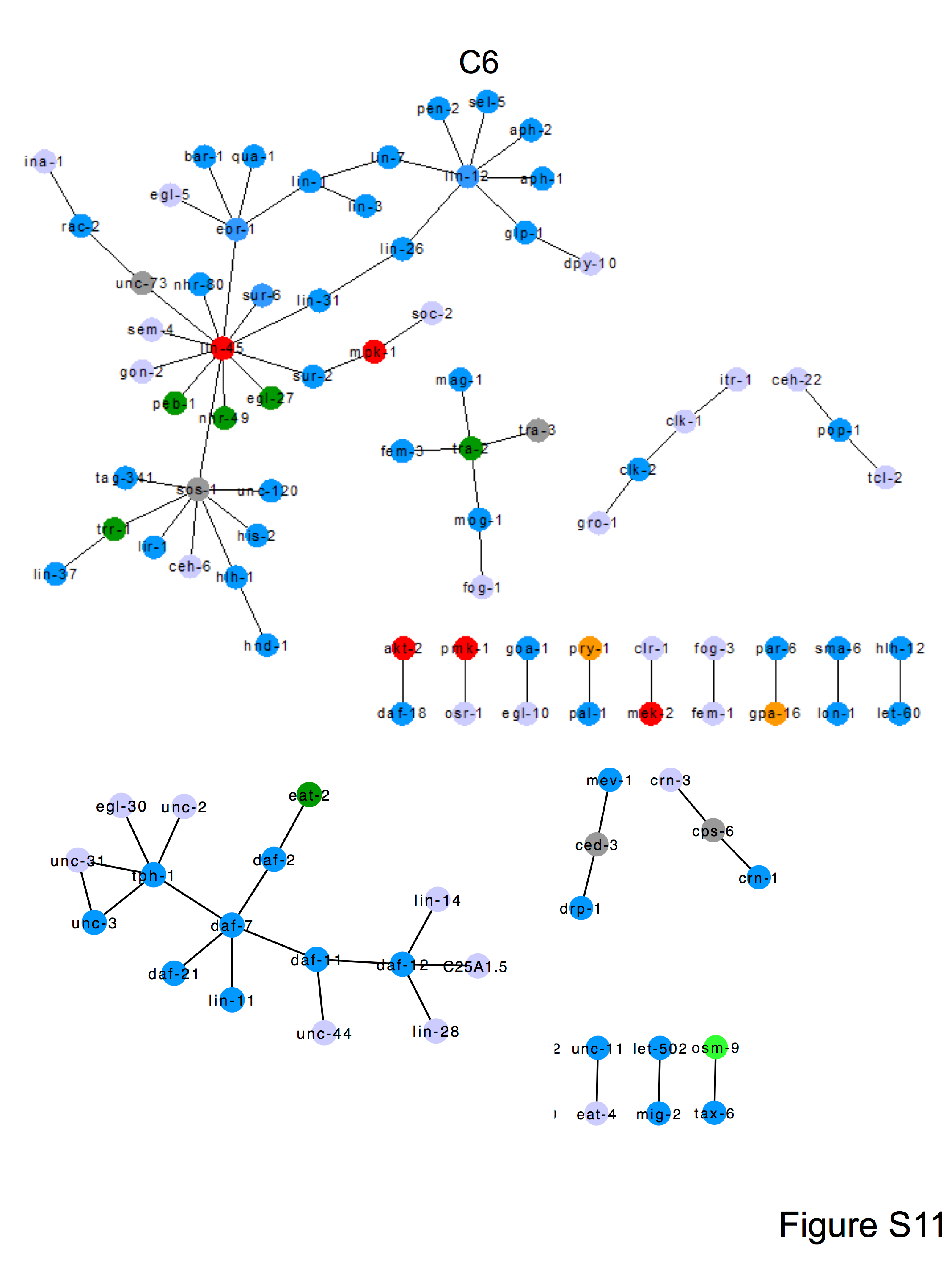

Supplement: S11 Fig — Refer to S5 Fig for the nodes and edges descriptions. (TIF) [file pcbi.1004738.s011.tif]

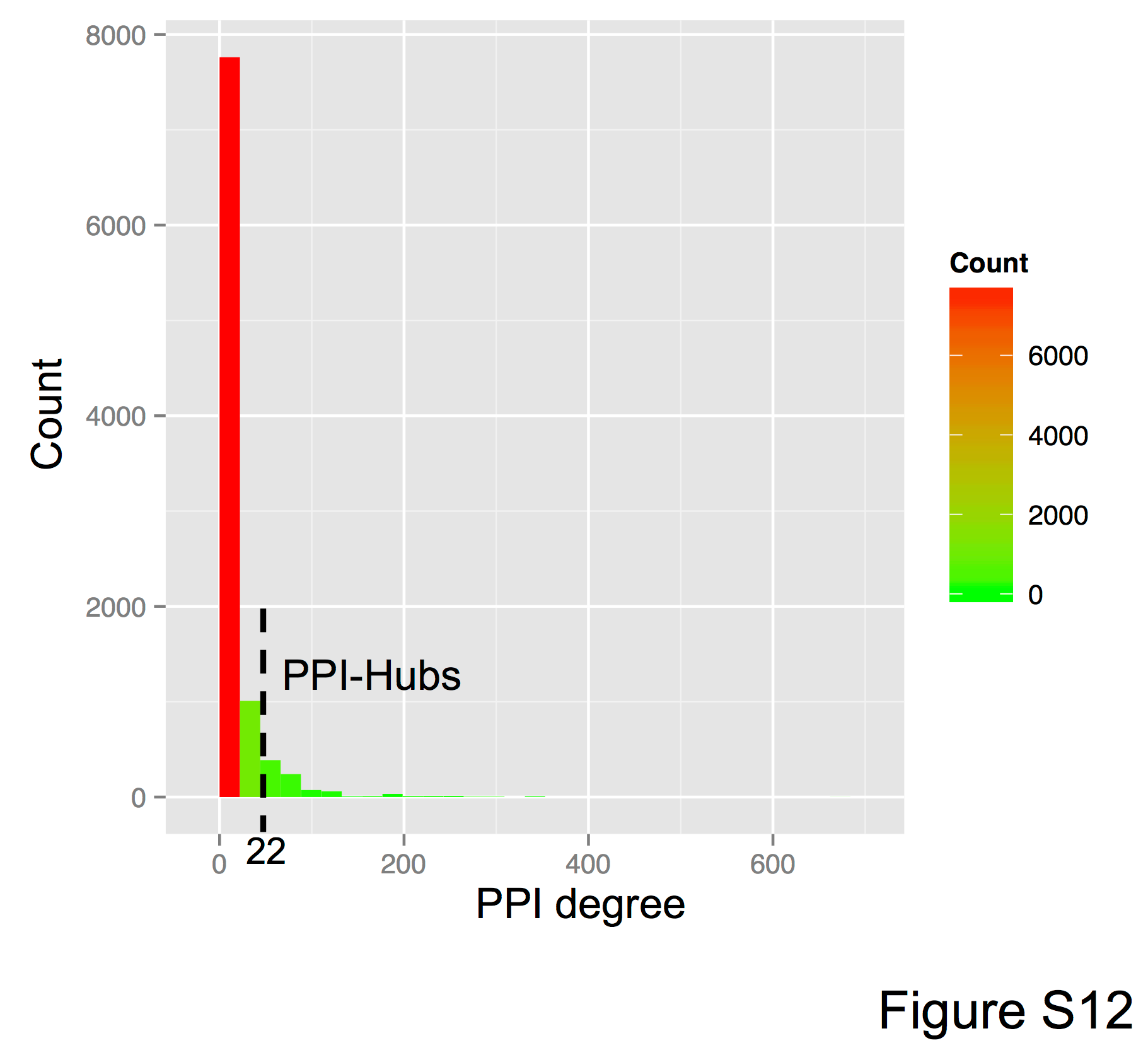

Supplement: S12 Fig — PPI-Hubs are identified as the top 20% most connected proteins in the PPI network indicated by a dashed line. (TIF) [file pcbi.1004738.s012.tif]

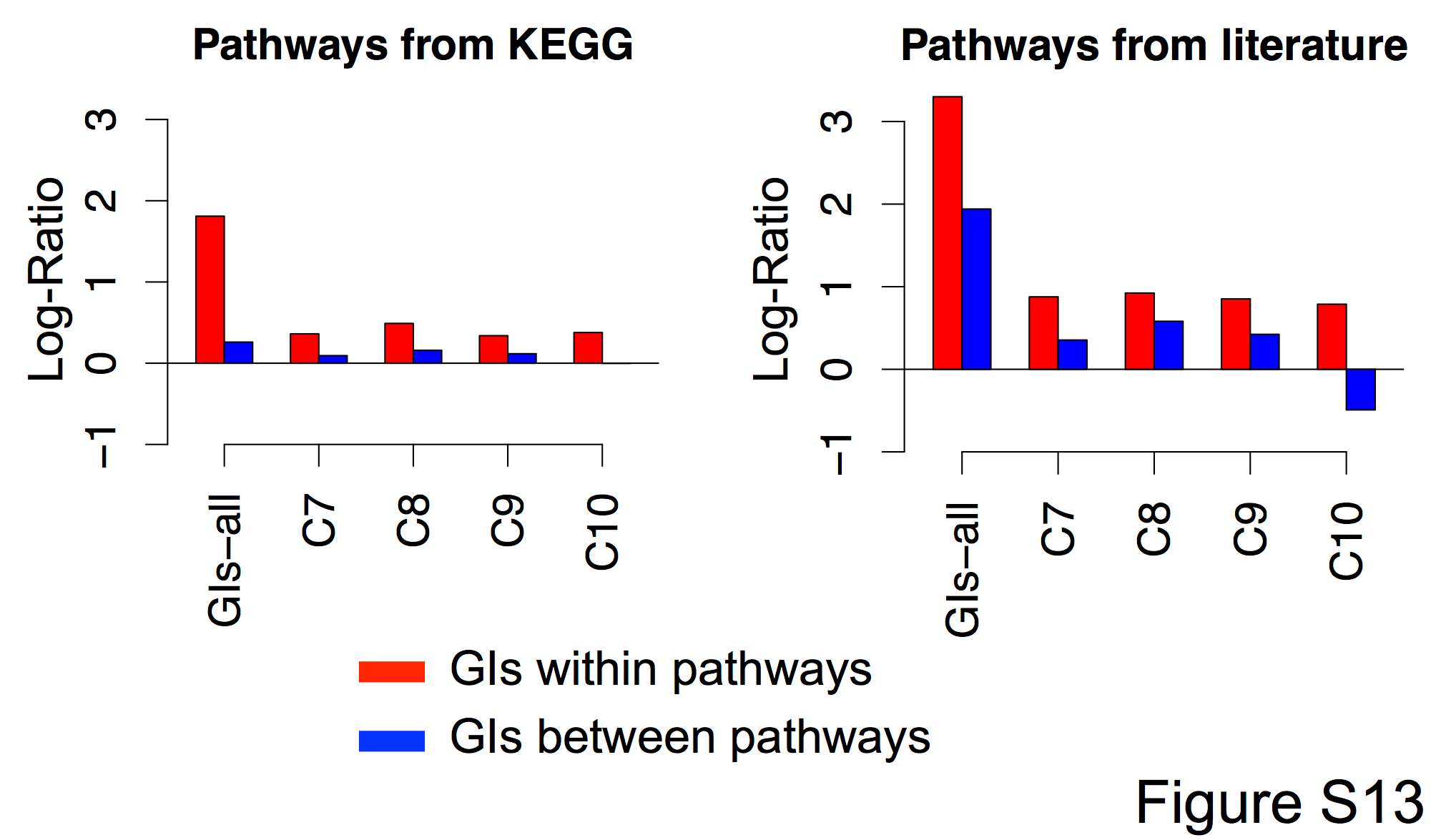

Supplement: S13 Fig — Log-Ratio scores for within-pathway (red bars) and between-pathway (blue bars) relationships observed between genes interacting through unselected GI classes (C7-C10) or present in GIs-all. A positive Log-Ratio score means that the frequency of within- or between-pathway GIs occurring in GI classes is significantly higher than the frequency of similar situations witnessed in relevant randomized GI networks with a probability of 0.99. (TIF) [file pcbi.1004738.s013.tif]

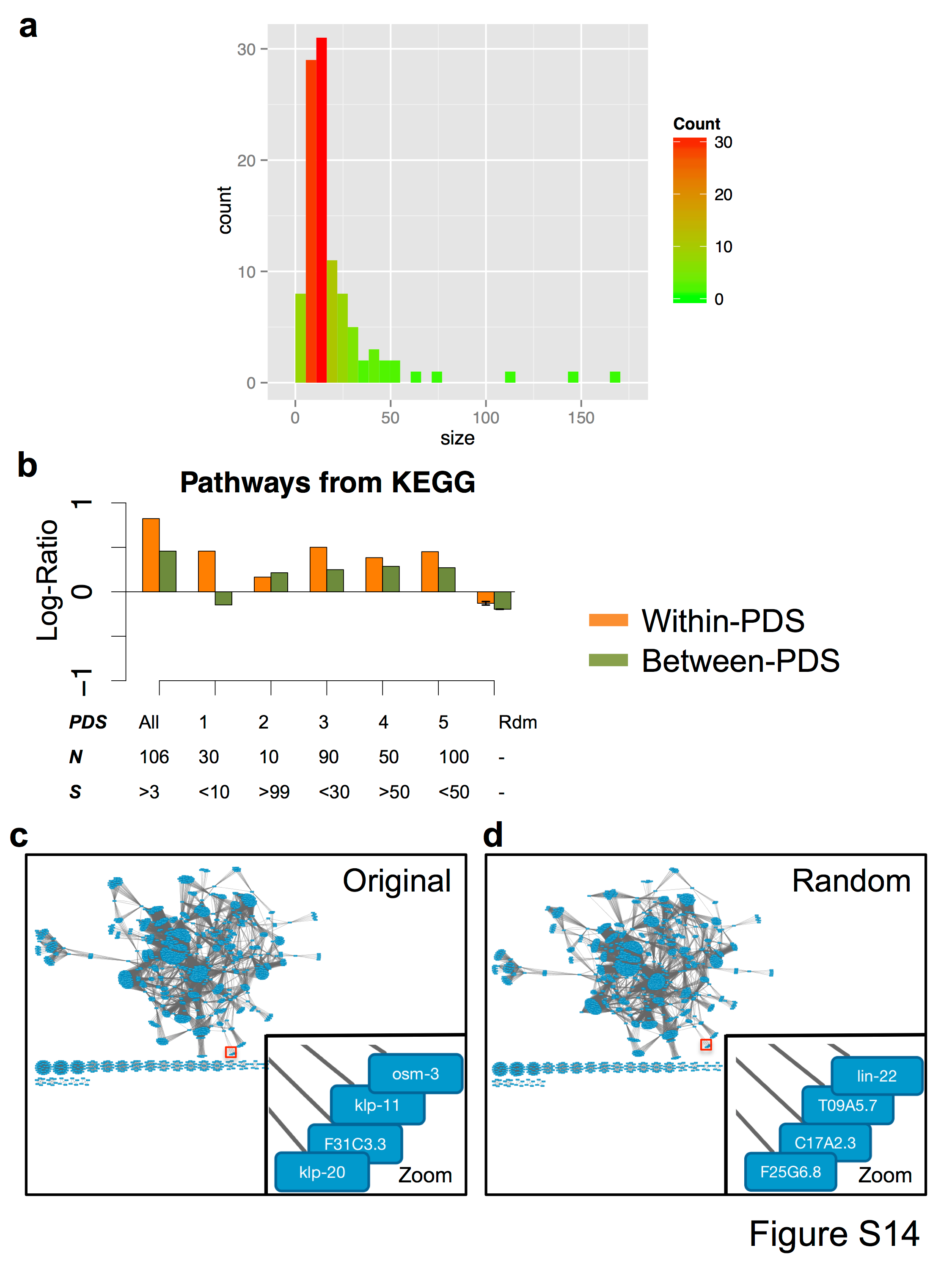

Supplement: S14 Fig — (a) Size distribution of 106 protein-protein interaction dense subnetworks (PDS). (b) Log-Ratio scores for within-PDS and between-PDS relationships occurring within-pathways. Different PDS networks were built by varying the number (N) and size (S) of the PDS. The original network "All" correspond to the union of PDS used for the study presented in Fig 6. Mean of Log-Ratio are indicated for Randomized PDS networks (Rdm) (n = 100) have the exact same topology than the original PDS network. Error bar indicate standard deviation of Log-ratio obtained across the 100 Rdm. (c-d) Depiction of the original and the random PDS networks topology. (TIF) [file pcbi.1004738.s014.tif]

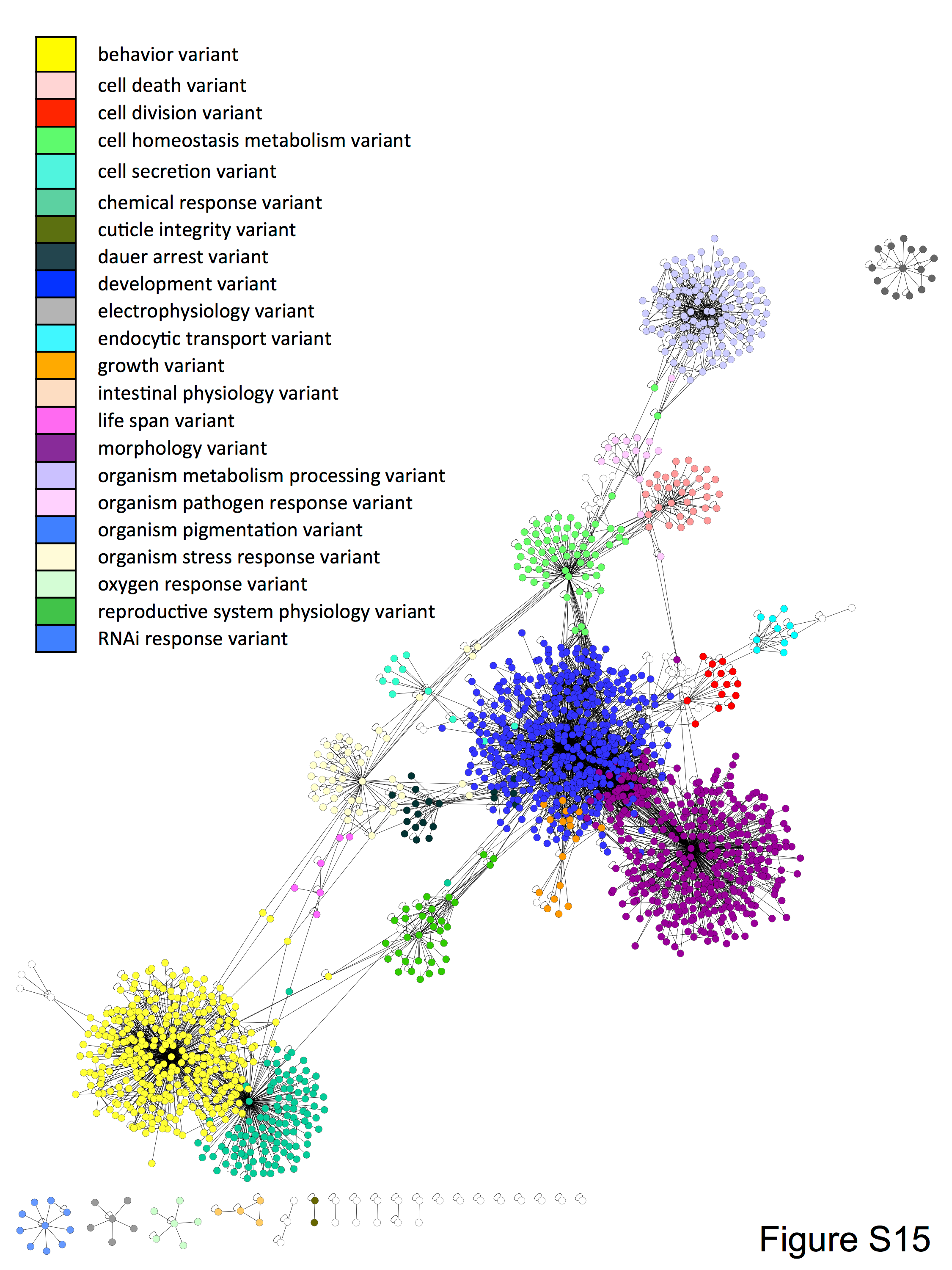

Supplement: S15 Fig — Phenotype IDs retrieved from WormBase (release WS220-bugfix) are represented by nodes and their hierarchical relationships represented by edges. Groups of phenotypes corresponding to the 22 most general phenotypes, and their first neighbors in the network were identified by different node colors. (TIF) [file pcbi.1004738.s015.tif]

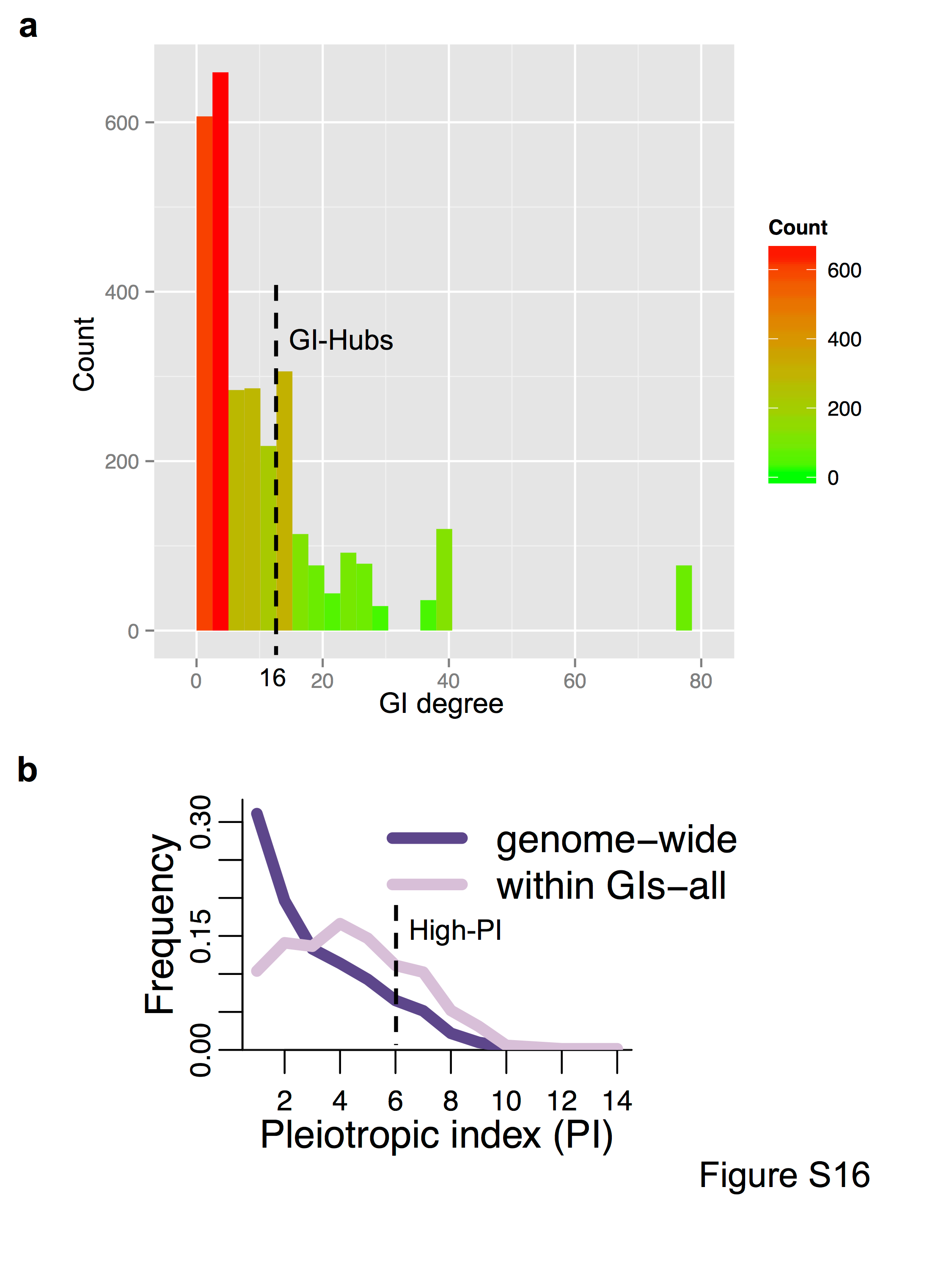

Supplement: S16 Fig — (a) Degree distribution for GI degrees in GIs-all. GI-Hubs, corresponding to the 20% GIs with the highest GI degrees, are located on the right side of the dashed line (GI degree ≥ 16). (b) PI distribution for all genes with PI > 0 in the C. elegans genome and for all interacting genes in GIs-all. High-PI genes, corresponding to the 20% genes with the highest PI, are located on the right side of the dashed line (PI ≥ 6). (TIF) [file pcbi.1004738.s016.tif]

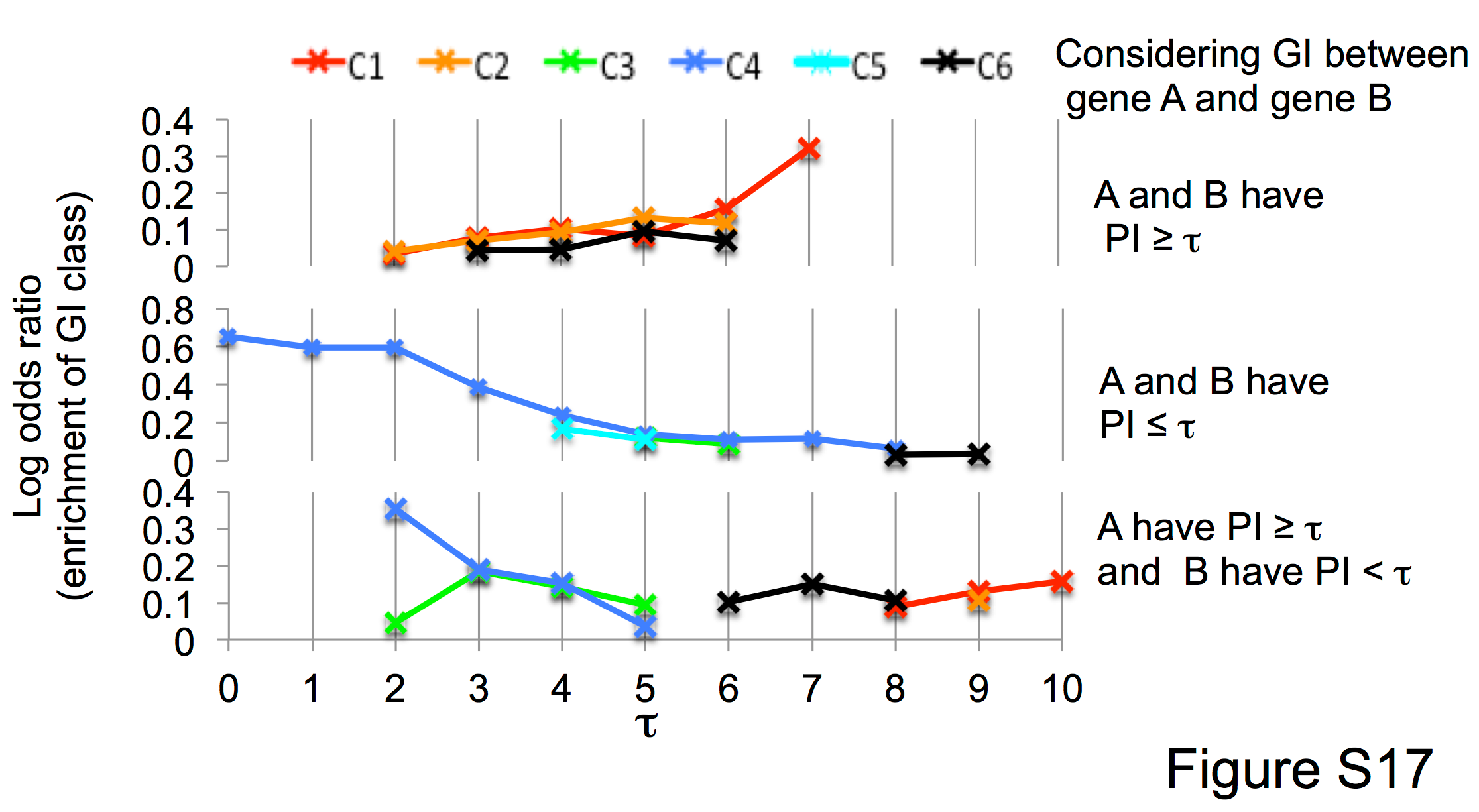

Supplement: S17 Fig — Log odds ratios of GI classes enriched in interactions between genes displaying the same range of PI higher or equal to a given threshold (τ) (upper panel); or lower or equal to τ (middle panel). GI classes enriched in interactions in which one partner (gene A) displays a PI ≥ τ and the other (gene B) display a PI < τ are also indicated. τ is indicated by the x-axis. Only significant enrichments of GI classes are indicated (Fisher’s exact test, P < 0.05). High log odds ratio indicates high enrichment of GI classes within or between indicated PI ranges. (TIF) [file pcbi.1004738.s017.tif]

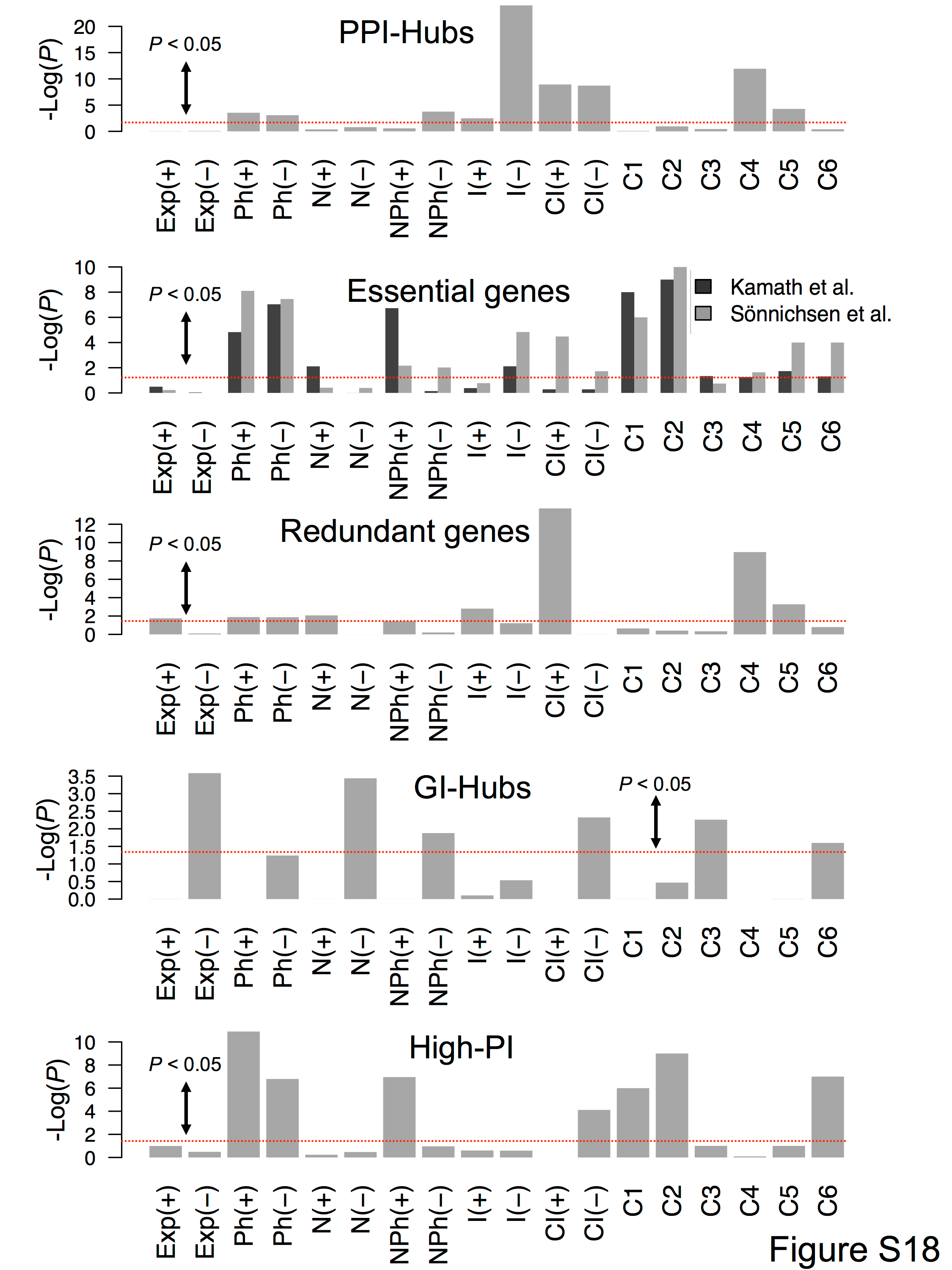

Supplement: S18 Fig — Enrichment in GI classes and GI groups of Genetic interaction-Hubs (GI-Hubs), redundant genes, protein-protein interaction Hubs (PPI-Hubs), Highly pleiotropic genes (High-PI) and essential genes. -Log of P-values from Fisher’s exact test are indicated. GI groups are associated to a positive value (+; above a threshold) or a negative value (-; below the threshold) for indicated attributes. Threshold values for each attributes are indicated S6 Table. (TIF) [file pcbi.1004738.s018.tif]

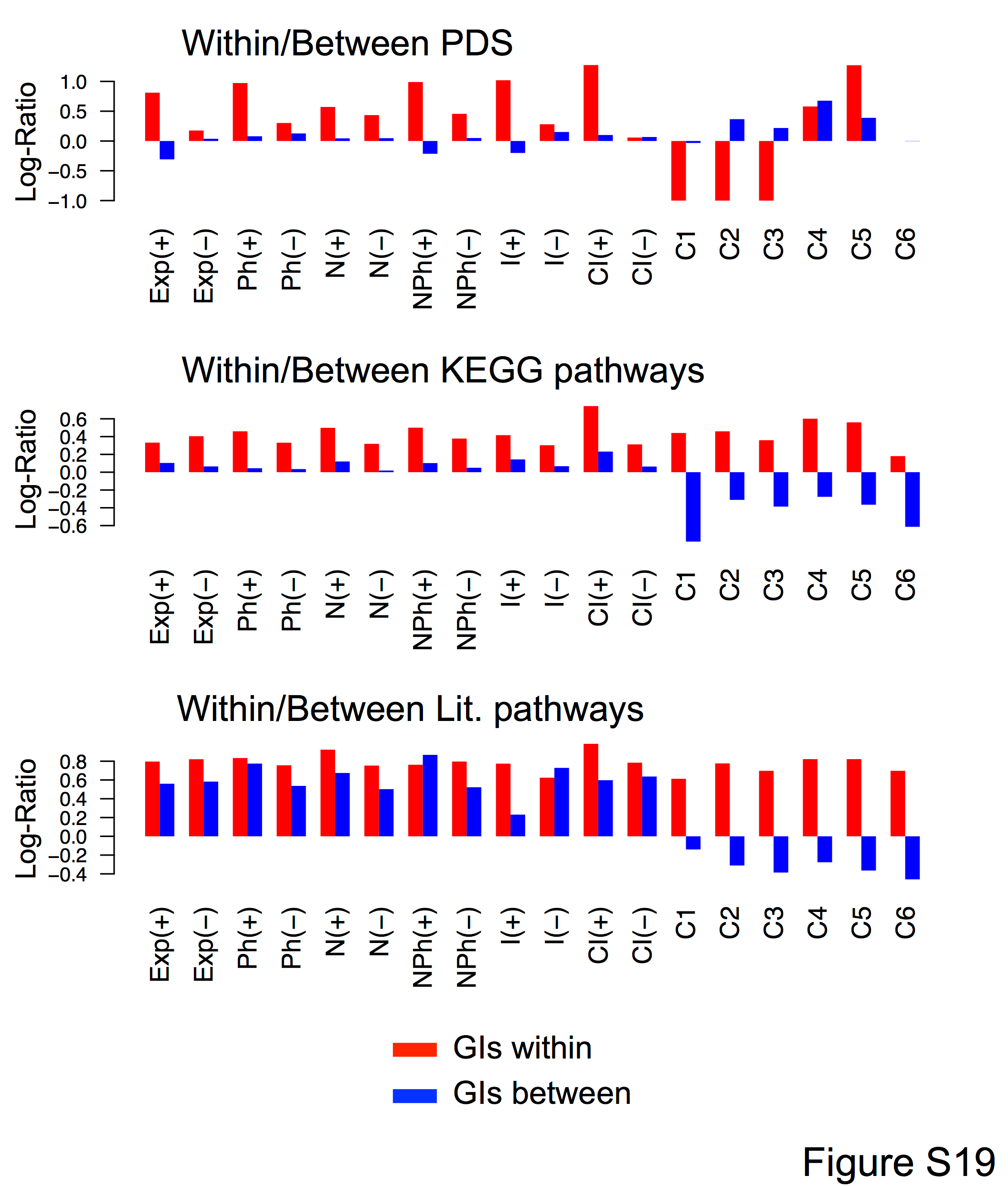

Supplement: S19 Fig — Log-Ratios profiles for GI classes and GI groups associated to a positive (+) or a negative (-) values for indicated attributes. Blue boxes indicate enrichment of biological characteristics for C4 and C5 GI classes and CI(+) GI group. (TIF) [file pcbi.1004738.s019.tif]

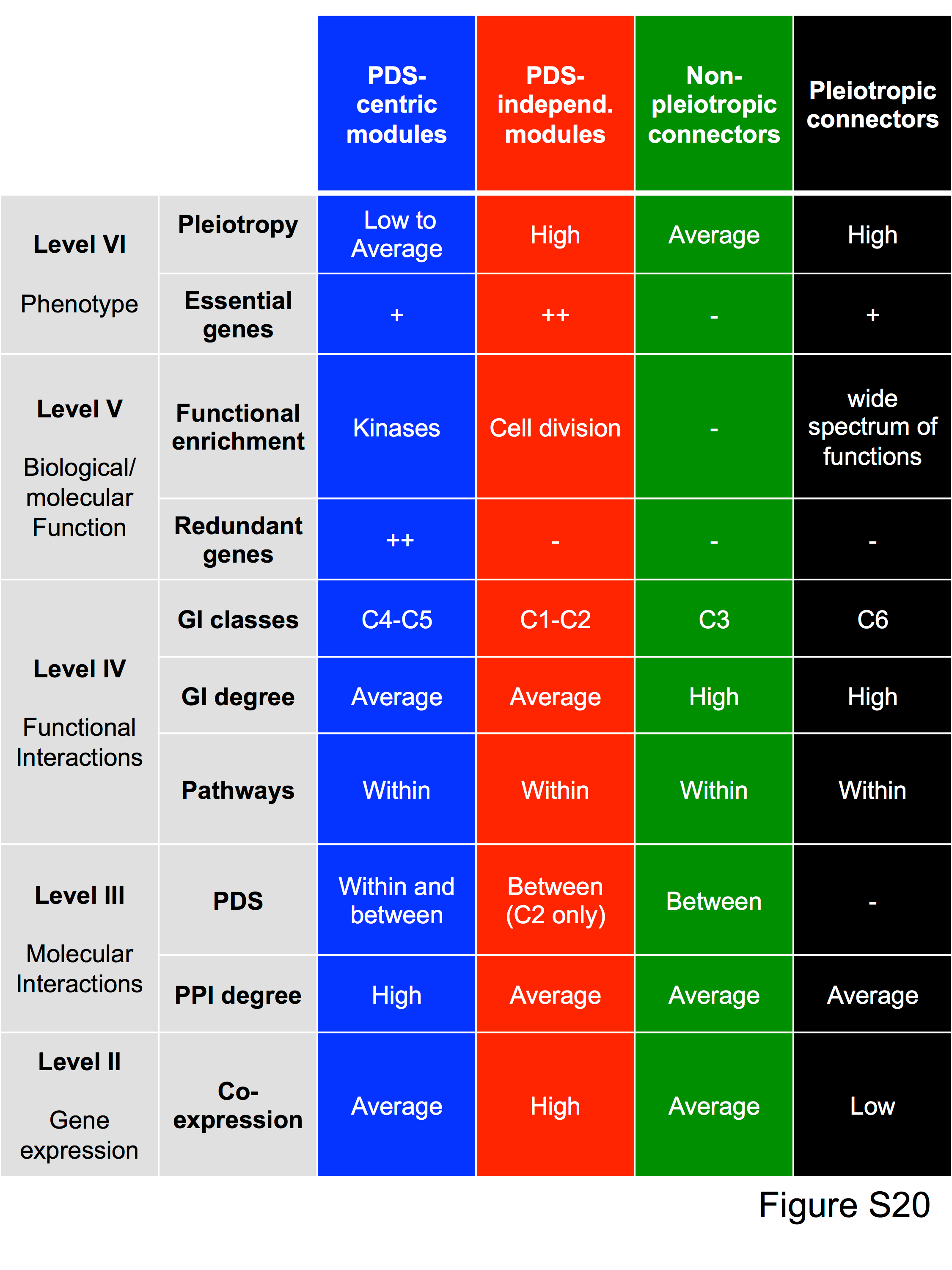

Supplement: S20 Fig — (TIF) [file pcbi.1004738.s020.tif]
