# Supplementary material for: Structural and Functional Characterization of a Caenorhabditis elegans Genetic Interaction Network within Pathways
Source: PLoS Comput Biol. 2016 Feb 12;12(2):e1004738. doi: 10.1371/journal.pcbi.1004738 (PMC4752231; doi:10.1371/journal.pcbi.1004738)
Supplement: S1 Text — (PDF) [file pcbi.1004738.s021.pdf]

Supplementary information

Robustness of GIs-all clustering into classes

We classified GIs from GIs-all using a cluster selection algorithm (Fig 1b; see Methods). Briefly, for each pre-selected cluster obtained by cutting the dendrogram at a certain height, we assessed the information content with the approximately unbiased bootstrap probability, which indicates how data support the clusters (see Methods). Secondly, we evaluated the classification performance of a logistic regression model using the cluster information content as a positive learning set and computed the performance with a leave-one-out cross-validation (LOOCV) test. A receiver operating characteristic (ROC) curve was then built following LOOCV results, which gave AUC scores between 0 and 1 (0.5 for a random prediction and 1 for a perfect prediction; S1 Fig). We judged that equivalently sized clusters with high AUCs were preferable over disparately sized clusters to ensure objective comparisons in further analysis. Thus, clustering the positive set of GIs identified 10 clusters and the six with AUC scores higher than 0.8 (C1 to C6) were subsequently analyzed to identify distinctive properties of GI classes (S1 Fig).

Impact of missing data on GI clustering

Despite the ever-growing amount of data coming from genome-wide studies, certain types of data, such as PPIs and phenotypical characterization of genetic alterations, still present very poor genome coverage. In addition, while all genes have expression data available, missing data is reported for the co-expression attribute for a certain number of genes that have expression values for less than 80% of the data used in our study. These data were used to compute five out of six of our attributes (see Methods; [1]). Before further characterizing the different classes of GIs, we asked whether the genomic signatures associated with each class might result from the sparse nature of genomic data used in the clustering. To evaluate this possibility, we calculated the percentage of GIs in each class missing expression data, PPI or phenotypical characterizations (S2 Fig). While a

certain amount of data were missing in each classes of GIs, detailed analysis revealed that missing expression, phenotype and/or PPI data did not significantly influence the distinctive properties observed for the six selected classes of GIs identified by our clustering technique. For example, GIs found in C1 and C2 segregate from C5 and C4 based on the absence of direct interaction between protein products of interacting genes (Int = 0; see Methods; Fig 1b) and the absence of an unexpectedly high number of common protein partners for these proteins within the PPI network (CI $P \geqslant 0.05$; see Methods; Fig 1b). At most 31.7% of GIs clustered in C1 and C2 lack PPI data (S2 Fig), suggesting that up to 31.7% of these interactions may segregate into C4 and C5 if using more complete protein interaction data. While this constitutes a significant portion of the GIs found in C1 and C2, these data suggest that the majority of C1 and C2 (more than 68%) are significantly distinct from C5 and C4 and consequently, that C1 and C2 constitute a real distinct class from C4 and C5.

C5 is composed of 51.7% of GIs expressing the phenotype enriched in their neighbourhood (*NPh* =1, see Methods; Fig 1b) and expressing proteins that share a large number of common interactors within the PPI network (*CI* < 0.05, see Methods; Fig 1b). These interactions could then segregate from C4 GIs based on the values of the *NPh* attribute (this value being null for C4 GIs). 60.4% of C4 GIs do not have any assigned phenotype upon genetic alteration. The lack of phenotypic data is then responsible for the null values of *NPh* for 60.4% of C4 GIs indicating that at least one of the interacting genes is not essential to any biological function assayed during genome-wide RNAi screening, or that the RNAi experiment used was not efficient enough to identify any phenotype. The cumulative false negative proportion (i.e. the frequency of genes having no phenotype association because of technical artefact) of genome-wide RNAi screenings reported in WormBase (release WS180) is impossible to estimate and may be responsible for the clustering of GIs in C4 instead of C5. C4 GIs are also distinguished from the second half of C5 GIs based on *I* and *CI* attributes. No missing PPI data are observed for C4 and C5 GIs suggesting that the segregation of these two clusters cannot be explained by missing PPI data.

C2 GIs were distinct from C1 based on the fact that C2 gene pairs do not express the common phenotype(s) enriched in their neighborhoods (*NPh* = 0, see Methods; Fig 1b). However, no missing phenotypical data is observed for C1 and C2 GIs, suggesting that different *NPh* values are a real distinctive property for differentiating C2 from C1 GIs. Missing expression data in C2 (S2 Fig) may also partially explain the lower average coexpression value observed in C2 when compared to C1, (Fig 1b).

C3 and C6 segregated from the other clusters based on the absence of a significant enrichment of similar phenotypes (*Ph* =1; see Methods; Fig 1b). However, none of the GIs in C3 and C6 lack phenotype data (S2 Fig), suggesting that the absence of a significant enrichment of similar phenotype is a real distinctive property of these two GI classes.

C3 and C6 distinguished themselves from the other clusters based on low coexpression values of interacting genes in C6 and on the lack of phenotype enriched in their neighbourhood ($N \geqslant 0.05$; see Methods, Fig 1b). Expression data are missing in 25.6% of GIs from C6 (S2 Fig). These data play also an important role in the computation of the *N* attribute since the neighbourhood of a gene is defined by its significantly coexpressed genes and the PPI partners of its protein product. This suggests that up to 26% of GIs from C6 may redistribute to C3, given more complete data sets. This also implies that the majority of C6 (more than 74%) retains distinctive properties when compared to C3.

Overall, these data show that missing expression, phenotype and/or PPI data cannot explain the classification of GIs-All in six classes with distinctive properties.

GI classes assembly into GI dense subnetworks

We tested whether GIs may assemble into GI dense subnetworks (GDS). To test this possibility, we identified GDS within GIs-all using the cytoscape “MINE”

plugin [2]. We identified 42 GDS (S3 Fig). For each GDS, we computed a monochromacity score (MS) adapted from similar study done on yeast GI network [3]. Positive and negative MS identified GDS enriched or depleted respectively in a given GIs class (see Methods). Clustering of GI classes using Euclidian distance based on MS revealed that GI classes tend to cluster by pair within GDS: C1 with C2, C4 with C5 and C3 with C6 (S3 Fig). Measurement of the enrichment of combination of classes using hypergeometric test also revealed that C1 is enriched alone or in combination with C2, C3 or C6 in a few GDS (S3b Fig). Similarly, C4 was found enriched alone or in combination with C5 (S3b Fig). These data suggest that while GDS number is too small to clearly measure a monochromaticity of GDS, GI classes tend to assemble in GDS in a biased manner.

We supported this assuption through measurement of the amount of overlapping genes in the selected GI classes. The rational behind this measurement is that GI classes that had a propensity to assemble in GDS together may also share common genes. To do so, we hierarchically clustered gene frequencies and used the resulting dendrograms as an indication of proximity between GI classes (S4 Fig). Two distance metrics were used to distinguish GI classes: Canberra distance was used to cluster GI classes when considering repetition of genes within classes (due to the potential involvement of genes in more than one GI). We used also Binary distance to cluster these classes base on gene composition without considering frequency. This study revealed that gene sets appear to be different between GI clusters (Binary; S3 Fig), and that the most connected genes are also largely different from one cluster to another (Canberra; S3 Fig). Interestingly, irrespective of the clustering method used, GI classes distributed in three groups, C1/C2/C3, C4/C5 and C6 based on gene overlaps (S4 Fig).

Enrichment in within- and between-PDS within pathways does not depend on the PPI network topology

We tested whether the size and the number of PDS may influence the enrichment of within- and between-PDS, within pathways. To do so, we analyzed

the distribution of PDS identified from our PPI network (S14a Fig), cut this distribution and generated several networks with different combination of small and large PDS. As seen in S14b Fig, these networks are composed of:

PDS-1: 30 PDS of less than 10 proteins

PDS-2: 10 PDS of more than 99 proteins

PDS-3: 90 PDS of less than 30 proteins

PDS-4: 50 PDS of more than 50 proteins

PDS-5: 100 PDS of less than 50 proteins

PDS-All: As a control we used the network composed of all PDS found in the multispecies PPI network.

PDS-Rdm: In order to strengthen this study, we used node permutation to generate 100 random networks displaying the same topology than PDS-all (same number of interactions, same number of PDS, same PDS size-distribution, same between PDS interactions; S14c and S14d Fig). These random networks are called PDS-Rdm (S14b Fig) and contain less than 3% overlapping edges with the PDS-all.

We then used these networks to assess the enrichment in within- and between-PDS interaction found within pathway as detailed in the Method section. This analysis showed that except for PDS-1 containing only PDS of less than 10 proteins, PDS-2 to -5 and PDS-all display a significant enrichment in within- and between-PDS interactions within pathways (S14b Fig). In addition, PDS-Rdm with the same topology than PDS-all did not display any enrichment neither in within- nor in between-PDS and even appears significantly depleted in these two kinds of interactions (S14b Fig). These data suggest that the enrichment of within- and between-PDS observed within-pathways did not depend on the topology of the PDS network but instead depends on the interactions themselves.

These data suggest that pathways and PDS are distinct functional modules.

They also suggest that pathways are composed of several PDSs and that proteins involved in the same pathway may be part of the same PDS or of different PDS. We confirmed these assumptions through a close examination of KEGG pathways and PDS as shown in S4 Table. We identified for each KEGG pathways and pathways from the literature genes involved in the same PDS (same-pathway & same-PDS) and genes involved in the same pathway and in different PDS (same pathway & different PDS). This study revealed that our assumption is exact and that up to 6 PDS may contain proteins involved in the same pathway and up to 4 proteins of the same PDS may be also involved in the same pathway. For example Vulval development is a pathway involving genes coding for proteins present in 5 different PDS. two genes LIN-45 and LET-60 are part of the same PDS, while 4 other genes, LIN-3, SEM-5, KSR-1 and LET-23 code for proteins that are part of 4 different PDS (S4 Table).

Distribution of GI classes within and between ranges of Pleiotropy

We analyzed the distribution of GI classes at different ranges of pleiotropy. To do so, we measured the enrichment of GI classes in GI subnetworks composed of genes within a similar PI range, either PI higher or equal to a certain threshold (τ) (upper panel, S17 Fig) or PI lower or equal to τ (middle panel, S17 Fig). Log Odds ratio and hypogeometric *P*-values are indicated in S5 Table. Interestingly, we found that C1, C2 and C6 GIs are enriched in interaction between genes with high PI (PI $\geq$ 7 for C1 and PI $\geq$ 6 for C2 and C6; upper panel, S17 Fig). On the other side, C4 is highly enriched in interactions between genes with low PIs (PI $\leqslant$ 2; middle panel, S17 Fig). C5 and C3 are enriched in interaction between genes with an average PI (4 $\leqslant$ PI $\leqslant$ 5 and 5 $\leqslant$ PI $\leqslant$ 6, respectively).

GIs may also link genes in different PI ranges. To test this possibility, we measured the enrichment of GI classes in sets of interactions linking genes with a PI higher or equal to a certain threshold (PI $\geqslant$ τ) with genes having a PI below this threshold (PI < τ). We tested this enrichment for every τ between 1 and 10 (lower panel, S17 Fig). These data showed that C1, C2 and C6 link

genes with average to high PIs (between 4 and 7; lower panel, S17 Fig) to genes with an even higher PI (from 6 to 10; lower panel, S17 Fig). This analysis also showed that C4 links genes with low PIs (from 0 to 2; middle panel, S17 Fig) to genes having an average PI (between 2 and 5; lower panel, S17 Fig). C3 appeared to do the opposite of C4, linking genes of average PI (from 5 to 6; middle panel, S17 Fig) to genes with low PI (from 2 to 5; lower panel, S17 Fig). C5 interactions appeared not to be enriched across ranges of PIs, suggesting that C5 interactions link genes mainly within an average PI range (from 4 to 5; middle panel, S17 Fig). Note that GI between genes that have very different PI will be considered to compute the Log Odds ratio more than once (meaning at different threshold (τ)). For example an interaction between a gene with PI=9 and a gene with PI=7 will be identified with τ = 9 and 8.

Altogether these data further characterize PDS-centric (C4-C5) functional modules composed of elements with average and low PIs, and PDS-independent (C1-C2) functional modules linking genes with high PIs. These data also further distinguish C3 from C6 connectors, with C6 linking genes from an average to a high level of pleiotropic and C3 interactions linking genes from an average to a low pleiotropic level.

References

1. Lee AY, Perreault R, Harel S, Boulier EL, Suderman M, et al. (2010) Searching for signaling balance through the identification of genetic interactors of the Rab guanine-nucleotide dissociation inhibitor gdi-1. PLoS ONE 5.
2. Rhrissorrakrai K, Gunsalus KC (2011) MINE: Module Identification in Networks. BMC Bioinformatics 12: 192.
3. Szappanos B, Kovacs K, Szamecz B, Honti F, Costanzo M, et al. (2011) An integrated approach to characterize genetic interaction networks in yeast metabolism. Nat Genet 43: 656-662.
